# Remotely Activated Nanoparticles for Anticancer Therapy

**DOI:** 10.1007/s40820-020-00537-8

**Published:** 2020-10-27

**Authors:** Luisa Racca, Valentina Cauda

**Affiliations:** Department of Applied Science and Technology, Politecnico di Torino, C.so Duca degli Abruzzi 24, 10129 Turin, Italy

**Keywords:** Anticancer therapy, Remotely activated nanomedicine, Stimuli-responsive nanoparticles, Physical stimulation, Radiofrequency, Nanoparticle-assisted ultrasound, Hyperthermia

## Abstract

Cancer has nowadays become one of the leading causes of death worldwide. Conventional anticancer approaches are associated with different limitations. Therefore, innovative methodologies are being investigated, and several researchers propose the use of remotely activated nanoparticles to trigger cancer cell death. The idea is to conjugate two different components, i.e., an external physical input and nanoparticles. Both are given in a harmless dose that once combined together act synergistically to therapeutically treat the cell or tissue of interest, thus also limiting the negative outcomes for the surrounding tissues. Tuning both the properties of the nanomaterial and the involved triggering stimulus, it is possible furthermore to achieve not only a therapeutic effect, but also a powerful platform for imaging at the same time, obtaining a nano-theranostic application. In the present review, we highlight the role of nanoparticles as therapeutic or theranostic tools, thus excluding the cases where a molecular drug is activated. We thus present many examples where the highly cytotoxic power only derives from the active interaction between different physical inputs and nanoparticles. We perform a special focus on mechanical waves responding nanoparticles, in which remotely activated nanoparticles directly become therapeutic agents without the need of the administration of chemotherapeutics or sonosensitizing drugs.

## Highlights

- The present review highlights the importance of remotely activated nanoparticles for anticancer purposes.
- For each physical input, we present its possible active synergy with several nanomaterials.
- We report examples and the mechanism of action when clarified.
- Clinical trials involving remotely triggered nanoparticles are discussed.

## Graphical abstract

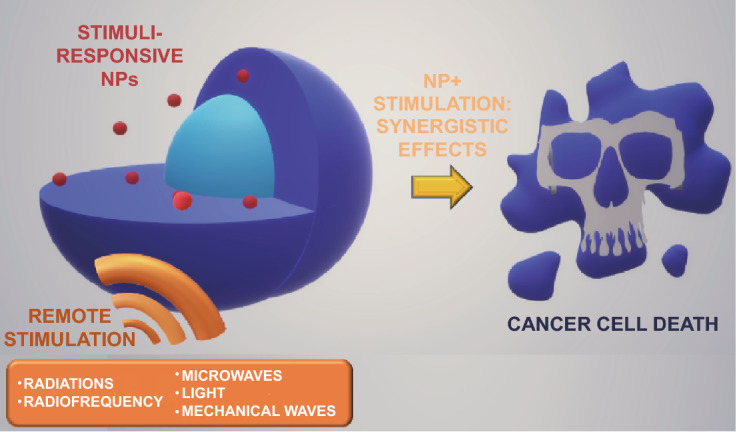

## Introduction

Cancer has nowadays become the second cause of death worldwide after cardiovascular diseases. Only in 2018 indeed, according to the statistics from the World Health Organization, 18.1 million new cancer cases and 9.6 million cancer-related deaths have been recorded [1]. Cancer cells are characterized by an uncontrolled growth and can invade other tissues even escaping the control of the immune system [2, 3]. Thus, the development of efficient techniques for the diagnosis and the early-stage treatment of this disease is essential, particularly in view of the continuous annual growth of new cancer cases in the coming years.

In the last years, the recently developed nanomedicine field has started to propose different solutions. Actually, nanomedicine is the application of nanomaterials, i.e., particles with a size range of 1–100 nm, for diagnosis, monitoring, prevention and treatment of diseases [4]. Additionally, the concept of “theranostic”, nanomaterials with both diagnostic and therapeutic properties, is emerging [5, 6]. Nanoparticles (NPs) have been widely employed in anticancer therapy, in particular for the delivery of cargo molecules, i.e., imaging agents, genes or chemotherapy drugs [5, 7–9], or alone, exploiting their intrinsic toxicity, e.g., related to the release of toxic species [10, 11]. NPs moreover could be decorated with chemical or biological coatings to improve their stealth properties and reduce their aggregation in biological fluids. In addition, they could be conjugated with targeting ligands to maximize their delivery to the desired target cells [12]. NPs could spontaneously accumulate into cancers thanks to the enhanced permeability and retention effect (EPR), because they can easily cross the tumor vasculature, characterized by the presence of large pores (< 2 μm) and the poor lymphatic drainage allows their retention, thus facilitating their therapeutic effect even in the absence of targeting ligands on their surface [13]. However, it was highlighted that a careful attention about NPs chemical, physical, and biological behavior has to be paid, as NPs could be responsible of numerous side effects. Therefore, also the sole NPs could be associated with negative outcomes as conventional cancer treatments [14, 15].

In the last years, several researchers have proposed a new anticancer therapeutic approach based on the conjugation of two different components, i.e., an external physical stimulation and a NP, which can be remotely activated by the stimulation. Both the stimulation and the NP themselves are administered individually at a harmless dose, however when administered simultaneously, their synergy results in the cancer cell death, also limiting the negative outcomes for the surrounding tissues [16–19]. Several physical stimulations have been employed so far for this purpose, such as radiations [17], radiofrequencies [20], microwaves [21], light [22], and mechanical waves [16]. Some of them otherwise are already used alone for anticancer therapy, e.g., radiation therapy. However, their action is often not focused on the tumor area and therefore also the surrounding healthy tissues could be seriously damaged [23, 24]. The mechanisms of action of the proposed synergy between the physical input and the nanoparticles have only sometimes been explained, while in other cases it is under debate or unclear. In general, the NP addition could amplify the effects of the physical stimulation, thus lowering the dose needed to obtain cell death by helping to focus its effects on the target site [17], or by absorbing the stimulus and releasing another form of energy to the surrounding medium [19, 23]. In other cases otherwise, the physical stimulation could trigger or improve the intrinsic cytotoxicity of the NPs [25].

**Fig. 1:**
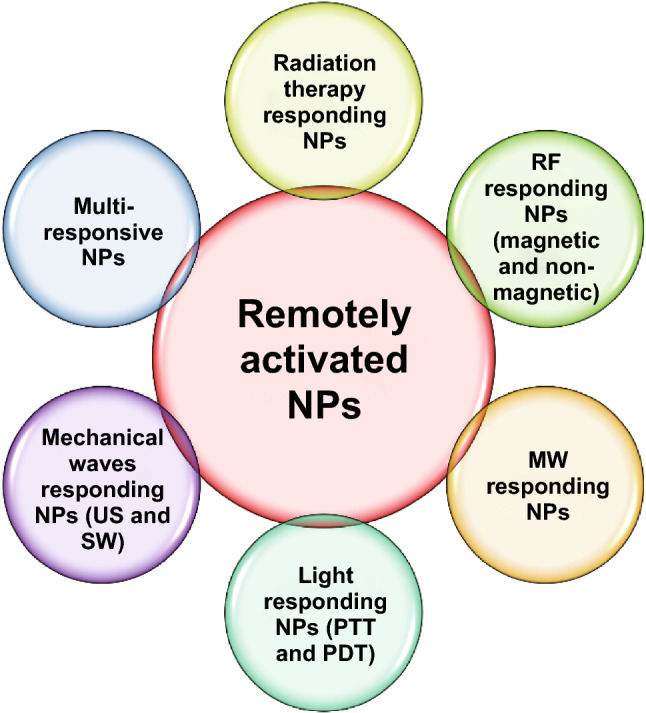
Overview of remotely activated NPs presented in this review. Radiation therapy, radiofrequency (RF), microwave (MW), light as photothermal therapy (PTT) and photodynamic therapy (PDT), mechanical waves as ultrasound (US) and shock waves (SW) responding NPs are presented, as well as NPs responsive to multiple simultaneous stimulations

Some significant reviews about externally triggered nanomedicines have already been published [26–29]. However, differently for these previous works, in the present review we focus on the active synergy between stimulations and NPs to achieve cancer cell death without the need for other adjuvants, e.g., drugs and sensitizers. Therefore, we highlight in this review only the effects produced by the active interaction between the stimulus and NPs that triggers the cytotoxicity in cancer cells. In particular, we exclude cases where the therapeutic action is not related to the activation of the NPs, whose role is often only to deliver the triggerable agent, i.e., drugs or other molecules, or for imaging purposes. We present several possible physical inputs as illustrated in Fig. 1, i.e., radiation, radiofrequency (RF), microwave (MW), light in photothermal therapy (PTT), and photodynamic therapy (PDT). We particularly focus on the most recent advancements related to mechanical waves, i.e., ultrasound (US) and shock waves (SW). Actually, the mechanical stimulation was often neglected in combination with the sole NPs without the addition of conventional sonosensitizers, while here it is the highlight of the present review. Additionally, the possibility to combine different and various physical stimulations to achieve an enhanced therapeutic effect is described. For each stimulus, its possible synergy with different types of nanomaterials is reported, proposing examples on the most recent results, discussing the possible mechanisms of action and finally providing a list of the clinical trials involving remotely activated NPs.

## NP-Assisted Radiation Therapy

**Fig. 2:**
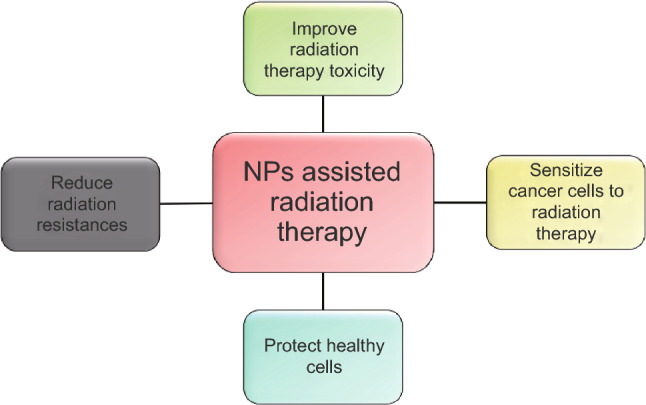
Schematic view on how the NPs can improve the efficacy of the radiation therapy in cancer cells

Radiation therapy, or radiotherapy, represents one of the conventional anticancer approaches [17], and it is employed to treat more than 50% of the whole cancer cases. It is based on the application of ionizing radiations, as gamma and X-rays, precisely focused on the tumor area. It is able to achieve cancer cell death by directly inducing DNA damages and through the production of reactive oxygen species (ROS) resulting from the oxidation of water and oxygen molecules [24]. ROS moreover displays a multifaceted role, because they are involved in several physiological processes and their level is kept under strict control. However, an excessive ROS production is cause of oxidative stress, affecting cellular components such as membranes and DNA, and altering cell signaling causing necrosis or apoptosis [30, 31]. Nevertheless, radiation therapy is associated with several drawbacks, such as the injury of the tissues in close proximity to the tumor and the development of resistances. Additionally, the treatment of hypoxic microenvironments, as several solid tumors, is challenging, because an increased radiation dose is necessary in this case to achieve cancer cell death and it could trigger severe outcomes for healthy tissues [24]. For these reasons, several researchers proposed strategies to both improve the toxic effects of radiation and reduce the side effects, as summarized in Fig. 2. The use of NP-assisted radiation therapy is a promising anticancer approach, and different materials were proposed for this purpose [24, 32].

A first possibility is to associate the radiation exposure with NPs able to enhance the radiation toxicity. High *Z* atomic number materials, as metal NPs, absorb and scatter indeed the radiation, generating also photoelectrons, Compton electrons, Auger electrons, and fluorescence photons. They have the advantages to reduce the damages to the neighbor tissues and enhance the radiation-associated injuries [17]. Gold, silver [17], gadolinium [33], bismuth [34], and platinum NPs [35] have been employed alone, or combined with chemotherapeutic drugs [17] or radionuclides and other radiosensitizers [36] to improve the radiation-mediated cellular damages. Otherwise, the addition of these elements to other nanomaterials, such as titanium dioxide (titania), confers them the capability to become responsive also to the radiation therapy enlarging their possible application as radiation-responsive NPs [17]. An example is represented by the X-ray photodynamic therapy explained below. The principal achievements regarding the use of these materials have been accurately summarized in several reviews [17, 35, 37, 38]. Hafnium oxide NPs in particular have already been approved for the European market and are involved in several clinical trials [24]. These NPs have demonstrated good radiation-enhancing properties not associated with injuries to the surrounding healthy tissue. Additionally, they have the remarkable property to amplify the radiation effects in a versatile way, leading to the potential use of these NPs for the treatment of various cancers. Actually, several other clinical trials on head and neck squamous cell carcinoma, liver cancer, prostate cancer, rectal cancer, and others are evaluating the efficacy of hafnium oxide NPs-assisted radiation therapy [39].

Also superparamagnetic iron oxide NPs have been proposed to improve the radiation-associated injuries [24]. Hauser tested iron oxide NPs on A549 lung cells carcinoma. Such NPs could react with hydrogen peroxide, generated as a consequence of the irradiation, releasing hydroxyl radical, improving the oxidative stress [40].

Also several non-metals have been employed to improve radiation therapy efficacy [17, 35, 38]. Selenium NPs were employed to enhance the radiation toxic effects on breast cancer cells by Chen et al. They observed that the combination of selenium NPs and the radiation therapy resulted in a decreased cell viability, a G2/M phase arrest and, in particular, an improvement in the ROS level with autophagy induction. These results were related to selenium ions release upon the radiation treatment and the subsequent enhanced ROS production [41].

NPs toxic features could also be exploited to sensitize cancer cells to radiation therapy. In this case, it is of a paramount importance that NPs satisfy this task without damaging the healthy cells. Zinc oxide NPs have demonstrated to be an optimal candidate for this purpose, because they are able to cause a tumor-selective cell death [10]. An example is the study of Meyer et al., where authors evaluated the cytotoxicity of zinc oxide NPs W/O irradiation on cancer cells and primary fibroblasts, highlighting the radiosensitizing properties of NPs only in cancer cells, perhaps related to the increased oxidative stress and cell cycle arrest caused by the addition of the NPs. However, the exact mechanism of action has still to be elucidated [42].

NPs could also be employed to deliver drugs able to protect the healthy tissues from the radiation toxic effects, limiting thus their negative outcomes, e.g., antioxidants that reduce the ROS damages [17], or to directly protect healthy cells thanks to their radioprotective intrinsic properties [24, 43, 44]. In this last case, cerium oxide NPs have demonstrated to both improve the radiation sensitivity and to possess radioprotective properties. Indeed, they have an antioxidant role in healthy cells, in which they act as ROS scavengers reducing DNA injuries, while in the acid tumor microenvironment they change behavior and contribute to decrease cell viability in combination with radiation therapy [45].

Another option is to target the radiation resistances of cancer cells, perturbing the relevant pathways with drugs delivered by NPs or using NPs that are able to affect these mechanisms [17]. Some groups suggest the use of nanomaterials able to alter the features of the tumor microenvironment that protect cancer cells from radiations, e.g., hypoxia. In this context, Abbasi’s group proposed the use of hybrid manganese dioxide NPs as radiosensitizers. They exploited in particular the reaction of NPs with the tumor metabolite H_2_O_2_ that results in oxygen generation. The reduced hypoxia was associated with a decrease in the radiation resistances and thus an improved antitumor effect [46].

Anyway, the use of conventional radiation therapy remains challenging for the treatments of pediatric cancers or tumors located near sensitive organs. Therefore, charged particle beams have been recently proposed as an alternative [47]. This approach is associated with reduced side effects for the neighbor healthy tissues, because energy is released only in the final track of the ion beam, and toxic effects could thus be limited to the region of interest tuning the initial energy of the beam. In addition, the administration of ion beams is associated to a more marked cytotoxicity, because of their increased ionizing properties. However, side effects in the regions in close proximity to the tumors could take place [47, 48]. Also in this case, several NPs have been proposed to maximize the injuries for cancer cells reducing the side effects for healthy tissues [48].

Since the clinical protocols are evolving [49] and new radiation sources are being born [47, 50], in the future innovative applications of the above-mentioned nanomaterials are going to be carried out. Similarly, the proposal of new NPs that work in synergy with the radiation therapy would certainly contribute to maximize the efficacy of this therapeutic approach.

## Radiofrequency Responding NPs

**Fig. 3:**
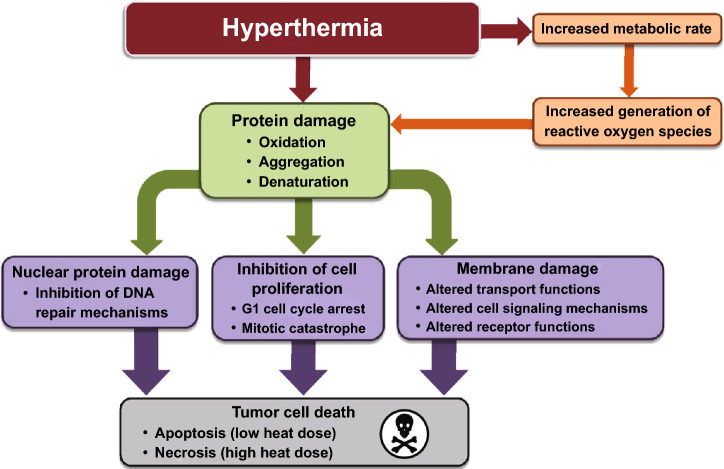
Cell injuries induced by hyperthermia. Reprinted under a Creative Common Licence CC-BY 3.0. Copyright 2020 from Ref. [52]

In general, the thermal treatment named hyperthermia is an historical valid option to achieve the killing of tumor cells [23]. Actually, when the temperature reaches values between 40 and 47 °C, several proteins can denature and form aggregates, impairing different pathways, such as the DNA repair and cell cycle progression (Fig. 3). These effects induce the cells to apoptosis. Instead, necrosis occurs when the temperature is over 50 °C [51, 52]. Cancer cells are more susceptible to the temperature rise because of their impaired vasculature that reduces their ability to face a change in the homeostasis conditions. In addition, elevated temperatures can also improve the perfusion of chemotherapies within tumor cells, enhancing the therapeutic outcomes [18].

There are several physical stimuli able alone to increase the temperature [52, 53]. However, hyperthermia is not tissue specific and it could cause severe injuries [23]. Besides, tumor cells can develop thermoresistance if the thermal treatment is repeated several times, thus decreasing its efficacy. For these reasons, hyperthermia is rarely involved in clinic alone, but it is proposed as coadjutant of other therapies, or with the addition of NPs that could contribute to focalize the heating within the tumor and limit the side effects [18].

Radiofrequency (RF) thermal ablation represents one of the hyperthermia-assisted anticancer approaches. In this particular technique indeed, hyperthermia is achieved by the application of an electrode that generates electrical currents into the tumor, improving the temperature through resistive heating [54]. The heat indeed is caused by the interactions between the electrons of the alternating current and the ions in the biological fluids (Na^+^, K^+^, Cl^−^) [55]. The stimulation is given through a needle that physically reaches the tumor and generates the high-frequency alternated currents necessary to produce heat. As a consequence, the temperature raises to around 50 °C and causes cell coagulative necrosis [56]. Since the heating is influenced by thermal conductance of the tissue, when the area of interest is carbonized, the ionic vibrations are limited, and thus the temperature rise is reduced [56]. However, it has been pointed out that the effective area of thermal ablation is very narrow, limiting the efficacy of this approach only for small tumors (< 3 cm diameter). Additionally, if the temperature reaches values over 100 °C, the consequent water evaporation and tissue dehydration cause a decrease in the electrical conductivity, reducing thus the thermal increase in the dehydrated areas [55].

The addition of several classes of NPs can contribute to maximize the therapeutic outcomes, supporting the temperature increase and limiting the damages for the healthy tissues. The use of NPs can furthermore help to improve the temperature only in the area of interest, actually where the NPs are localized. In this case, the needle insertion is not required, because only the tumor area is selectively heated by the specific response of the NPs, internalized into the cells or present in the tumor microenvironment. Several classes of magnetic and non-magnetic NPs have been employed in synergy to a RF application, exploiting their ability to improve the temperature for anticancer purposes. Typically, the application of an external source generates RF waves with a frequency range of 10 kHz–900 MHz. The mechanism under which this heating takes place is multifaceted and depends on both the RF source and the nanomaterials used. Actually, two types of sources are generally recognized: (1) inductively coupled device that produces an alternating magnetic field (MF) inside a solenoid and (2) capacitively coupled device that produces an alternating electric field between parallel-plates [57]. Magnetic NPs, e.g., iron oxide NPs, could be stimulated by the MF, whereas non-magnetic ones, e.g., gold NPs, seem to be more responsive to electric fields [58]. For these reasons, magnetic and non-magnetic NP-mediated RF-hyperthermia are sometimes identified as two different approaches, e.g., as proposed by Beik, nanomagnetic hyperthermia (NMH) and nano-radio-frequency ablation (NaRFA) [59].

Here we distinguish between magnetic and non-magnetic NPs presenting a brief overview of both the mechanisms with some examples.

### Magnetic NPs

The tumor heating with the combination of MF and magnetic NPs is emerging as promising anticancer approach, named sometimes magnetic fluid hyperthermia (MFH) [60], nanomagnetic hyperthermia (NMH) [59] or magnetic hyperthermia therapy (MHT) [61]. It was indeed observed that the synergy between these two components is able to heat the tumor more efficiently than other techniques [18]. The effectiveness of this approach is related to the specific absorption rate (SAR) value. This depends on the features of the MF applied, on the size and composition of the NPs and the properties of the solvent [60].

**Fig. 4:**
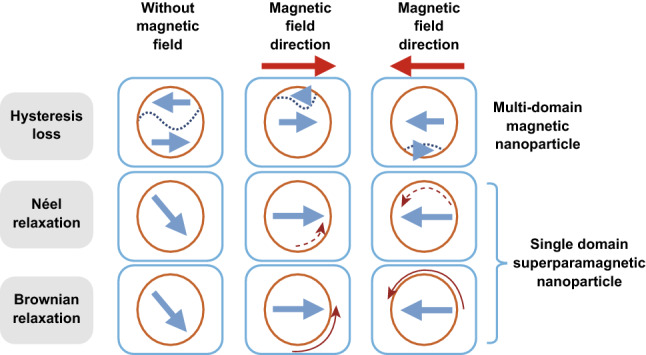
Magnetic NPs interaction with magnetic field (MF). NPs are displayed as orange circles with blue arrows indicating the magnetic domains. Dark red arrows represent magnetic moment direction movements (solid) or changes (dashed). Reprinted under a Creative Common Licence CC-BY 4.0. Copyright 2020 from Ref. [18]. (Color figure online)

The temperature increase due to the synergy of magnetic NPs and MF is caused by different mechanisms, as depicted in Fig. 4. The first one, hysteresis loss, verifies when all the different magnetic moments possessed by the NPs continuously align following the alternating MF applied and energy is released improving the temperature of the environment. Otherwise, when the size of the NPs is below 128 nm, a single magnetic domain remains, giving them superparamagnetic properties, and in this case Néel or Brownian relaxations prevail. Néel relaxation refers to the fast realignment of the magnetic moments under a MF that, contrasting with the NP crystalline structure, generates heat. Brownian relaxation instead verifies when NPs try to align with the MF and the friction caused by their movements improves the temperature of the medium [18].

In addition, magnetic NPs could be employed as contrast agent for computed tomography and magnetic resonance imaging, allowing thus both therapy and diagnosis [18, 62]. The outcome of the synergy of magnetic NPs and MF is influenced by the features of both [18]. It was demonstrated that NPs with different sizes could heat differently under a MF input [63], but other factors, as the morphology and anisotropy of the NPs and the presence of a coating agent, as well as the viscosity of the medium, influence the heating efficacy and thus have not to be underestimated [61].

Iron oxide NPs, classified as magnetite, maghemite or haematite according with their structure, are the most employed nanomaterial stimulated with MFs, especially magnetite and maghemite NPs [63]. Furthermore, iron oxide NPs could contribute to cell death not only improving the temperature in synergy with a MF, but also triggering some non-thermal effects. Hemery and co-workers compared the cytotoxic power of iron oxide NPs with a different morphology, actually monocore NPs, thus nanospheres, versus multicore NPs, whit a nanoflower shape, discovering that multicore NPs caused an increased cell death in synergy with an alternating MF with respect to the monocore ones. This is explained by the higher SAR of the multicore NPs, and additionally showed a higher internalization into glioblastoma cells, causing thus a higher heat generation under a MF stimulation. Additionally, these NPs could cause mechanical stress, i.e., membrane deformation, and release a high amount of Fe^2+^ ions, that could cause in turn oxidative stress through Fenton reaction [64].

Cobalt and nickel also exhibit the same properties of iron oxide, but they are not essential elements for the body, and thus they accumulate and cause toxicity. Additionally, they are more susceptible to oxidation [65]. However, they were both proposed in combination with other materials for therapeutic application in synergy with MF [63], as iron oxide [65], or with semiconductors, as stannic oxide, titanium dioxide [66], and zinc oxide [67], but also other elements could be combined for the same purpose. Jadhav presented a remarkable study about the doping of manganese zinc ferrite NPs with the rare-earth gadolinium. They indeed substituted gadolinium metal cations in spinel ferrites, altering the magnetic and electric properties of the NPs. They evaluated the influence of the chemical properties of the doped NPs related to the gadolinium concentration, discovering that this influences the structural, colloidal and magnetic properties of the NPs. However, these NPs possessed improved magnetic properties and heating performances. The NPs resulted to be non-toxic for human lung carcinoma cells alone, but highly cytotoxic in synergy with the MF stimulation, electing such doped NPs as promising anticancer agent for magnetic hyperthermia [68].

An important problem related with the use of these nanomaterials is the possible magnetic interactions occurring between the NPs that causes agglomerations, alters their responses to the MF, and limits their use for in vivo investigations. This evidence could be solved with the shielding of the NPs [65]. However, it was highlighted that the coating could alter the NP intrinsic magnetic properties, and thus it is of a paramount importance to prevent their loss to achieve successful results. The main purposes of these modifications are to increase the NPs biocompatibility and colloidal stability, enhancing at the same time their properties, e.g., their efficiency of heating. Silica [12, 69], gold [70], copper, palladium, and other metals have been extensively investigated as NPs inorganic shielding [65, 71]. The coating with gold is particularly attractive because the gold layer improves the chemical stability and biocompatibility of the magnetic NPs. It further allows to exploit the optical and radiosensitizing properties of gold to achieve tumor ablation with the combination of different external physical stimulations [62].

Also organic materials, such as citrate, dextran, sugars, and several polymers, as polyethylene glycol (PEG) [72], have been proposed for the same purpose [71]. Kandasamy et al. tested the influence of different coating molecules at different densities on the magnetization of iron oxide NPs, furthermore evaluating the synergy with a MF stimulation on liver cancer cells. They highlighted that only some organic-coated NPs displayed high magnetization values and were characterized by different thermal responses under MF stimulation [73].

Magnetic NPs in synergy with MF stimulations were also employed for the delivery of chemotherapeutic drugs to cancer cells, improving their therapeutic efficacy and reducing the side effects. This result could be obtained conjugating the NPs with the drug, and concentrating the NPs, and thus the drug, into tumor area through a remote magnetic control, as revised elsewhere [62, 74].

### Non-magnetic NPs

Several non-magnetic NPs have been employed in synergy with RFs to cause hyperthermia in tumors. Among others, gold NPs are particularly employed for this purpose. These NPs indeed have demonstrated to improve the temperature and the cell death when associated with a RF input, but the exact mechanism is under debate, and three possibilities have been presented.

The first mechanism proposed is the Joule or inductive heating. Actually, gold NPs could act as conductors under the RF stimulation. Therefore, the electric field penetrates into the NPs, but there is a resistive dissipation within the NPs that causes the temperature increase by heat dissipation. However, experimental observations demonstrated that this explanation is not correct, and thus the Joule heating has been rejected as possible mechanism to explain this synergy [57]. Nonetheless, magnetic heating and electrophoretic heating have been proposed in the last years as the most probable alternatives to explain RF heating of gold NPs. Magnetic heating refers to the capability of magnetic NPs to generate heat under a magnetic stimulation. In the case of gold nanomaterials, they could become magnetic after a chemical oxidation [58]. Electrophoretic heating instead is due to the movement of charged species on the gold NPs surface because of the variations of the electric field: this effect causes the oscillation of the NPs and generates heat through mechanical friction [20, 58]. The predominance of a mechanism over the other is probably associated to the features of the experimental setup, regarding both the RF source and the NPs [20]. Cardinal et al. reported the combination of gold NPs with a non-invasive RF generator to ablate tumors and avoid the side effects associated to the conventional invasive probes employed in the RF tumor ablation therapy. In vitro cell tests demonstrated that these NPs, upon the exposure to the RF field, improved the temperature achieving cell death. Additionally, the in vivo study confirmed the promising results obtained in vitro [75]. The NPs size, shape, concentration, and aggregation may affect the RF heating. In this context, Amini et al. compared the influence of the surface chemistry to tailor the behavior of gold NPs and nanorods under a RF stimulation. Comparing pristine with respect to cetyltrimethylammonium bromide (CTAB), citrate, and PEG-coated gold nanostructures heated under the RF irradiation, they observed a different rate of temperature increase due to the presence of the different shapes and coatings. Additionally, PEGylated NPs and nanorods coincubation with human pancreatic carcinoma cell line resulted in a decreased cell viability after the RF exposure, confirming the synergism between gold nanostructures and RF [76].

Also carbon-based nanomaterials have been proposed to enhance the heating consequences of a RF irradiation [77]. Gannon et al. highlighted the ability of single-walled carbon nanotubes to produce heat under a RF stimulation. Three cancer cell lines, HepG2, Hep3B, and Panc-1, were incubated with different concentrations of nanotubes functionalized with the Kentera biocompatible polymer, actually a polyphenylene ethynylene-based polymer, and exposed to a RF stimulation. A 100% cytotoxicity was obtained for all the three cell lines when incubated with the higher concentration of nanotubes (500 mg L^−1^) after 2 min of RF exposure due to the temperature rise caused by the addition of the nanotubes [78]. Bijukumar otherwise tested graphene for RF-hyperthermia in three-dimensional (3D) culture of liver cancer and in an in vivo model. The NPs were furthermore conjugated with the transferrin ligand to improve the uptake by liver cancer cells improving thus the hyperthermia effects as observed in vitro and in vivo [79]. However, carbon is often associated with other materials with magnetic properties, as iron oxide and cobalt, to obtain hybrid NPs with an optimal response to the magnetic component of the RF stimulation [59]. Furthermore, there are several examples of hybrid carbon structures [80, 81]. These nanohybrids are mainly involved in investigations where two or more physical stimulations are combined to achieve tumor ablation [82, 83].

**Fig. 5:**
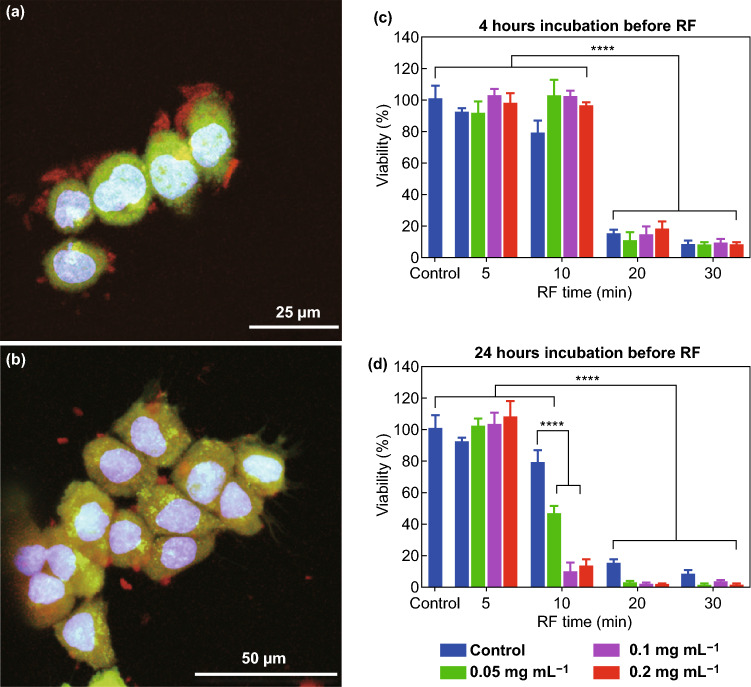
**a** Confocal images of HepG2 cells incubated with PSi NWs 4 h and **b** 24 h. **c** HepG2 cells viability upon the incubation with different concentrations of PSi NWs for 4 h and the further RF irradiation. **d** HepG2 cells viability upon the incubation with different concentrations of PSi NWs for 24 h and the further RF irradiation. Results are shown as mean ± SD ****p < 0.0001. Adapted with permission from Ref. [85]

Other nanomaterials have also been tested for this purpose. Silicon NPs possess good properties of biocompatibility and biodegradability. Additionally, their intrinsic capability to amplify the effects of different physical stimulations allows their use for several therapeutic approaches. Silicon NPs indeed demonstrated to possess a higher heating rate than gold NPs [84]. Gongalsky et al. evaluated porous silicon nanowires (PSi NWs) combined with a RF stimulation (40 W) as anticancer approach. PSi NWs were not internalized into HepG2 cells at the considered time points (4 and 24 h) as shown in Fig. 5. Observing the cell viability upon the incubation with different non-toxic concentrations of PSi NWs, for 4 or 24 h, and treated with RF for 0, 5, 10, 20, and 30 min (panels c and d), it was highlighted that the decrease in cell viability was significantly exasperated after 24 h of incubation, where also 10 min of RF exposure became sufficient to achieve cell death. The authors explained the cell mortality with the degradation of PSi NWs outside the cells, the result in the formation of silicic acid ions. Such ions induce the heating when exposed to the RF field, probably because the ions oscillation cause energy dissipation and Joule heating of the solution [85].

Another example is reported by Ashokan. Here, Fe^3+^-doped calcium phosphate NPs were employed for both imaging and therapy. The iron doping causes a characteristic augmented ionic conductance which increases the dielectric loss of the NPs under the RF stimulation. This effect provokes a temperature rise responsible of the observed necrosis [86].

Furthermore, quantum dots have been proposed in synergy with RF. Quantum dots are very small semiconducting nanoparticles (< 7 nm diameter), as cadmium-selenide or indium-gallium-phosphide, with a single material or multiple core–shell structure. They are frequently used for imaging and diagnosis [87]. Glazer investigated an in vitro model of mixed cancer cell populations to determine the synergy between RF cadmium-selenide and indium-gallium-phosphide quantum dots or gold NPs, all conjugated with CD225 antibody, to target epidermal growth factor receptor (EGFR). Cell mortality, following the coincubation with all the three types of nanostructures and RF stimulation, was increased only in human pancreatic carcinoma cells, that overexpress EGFR, while was less relevant in human breast carcinoma cell line expressing a lower amount of EGFR. Cell mortality was related to the temperature increase caused by all the considered nanostructures under a RF field [88].

## Microwave Responding NPs

Microwave (MW) thermal therapy is based on the application of electromagnetic waves with a frequency in the range from 915 MHz to 2.45 GHz and an energy in the MW energy range (300 MHz to 300 GHz) [89] to achieve tumor cell death through overheating, with the generation of temperatures higher than 150 °C [90]. Under this stimulation, the dielectric hysteresis verifies. Actually, polar molecules, as water, already present in the tissues, are forced to realign with the oscillating field, enhancing the kinetic energy and thus the temperature of the irradiated tissue [89]. Moreover, the use of MWs for thermal ablation presents several advantages if compared with other remote physical stimuli. MWs indeed possess the outstanding properties of efficient propagation through the body and effective heating of tissues characterized by a low thermal conductivity, allowing a more efficient heating and tumor ablation [54, 89].

The MW stimulation is triggered to the focal area thanks to an applicator directly inserted into the tumor. This means that for each treatment the applicator needs to be physically introduced into the body, with the risk of bleeding due to repetitive applications. Additionally the heating treatment is not tissue specific, and thus also areas neighboring the zone of interest could be subjected to an uncontrolled temperature increase, resulting in coagulative necrosis of healthy cells [21], inflammations and furthermore an increased risk of metastasis [91]. On the other hand, the inefficient heating of the tumor areas near the focal region, where the MW applicator is applied, is responsible of the recurrences [21]. For these reasons, some researchers proposed the use of NPs able to amplify the MW effects, while reducing the MW dose, in order to improve the therapeutic efficacy of this anticancer treatment and reduce the side effects [21].

A possibility is to employ nanomaterials that, in synergy with MWs, improve the temperature absorbing energy [21] and may promote a Joule heating effect [92]. Additionally, it is reported that some nanotools, e.g., iron oxide NPs and carbon nanotubes, are able to produce shock waves under a MW input, provoking a mechanical stimulation of the cells [93, 94]. The presence of these NPs moreover tunes both the thermal and electrical conductivities of the tissue, increasing the thermal ablation efficiency [95], but the operating mechanism is still unclear [96].

**Fig. 6:**
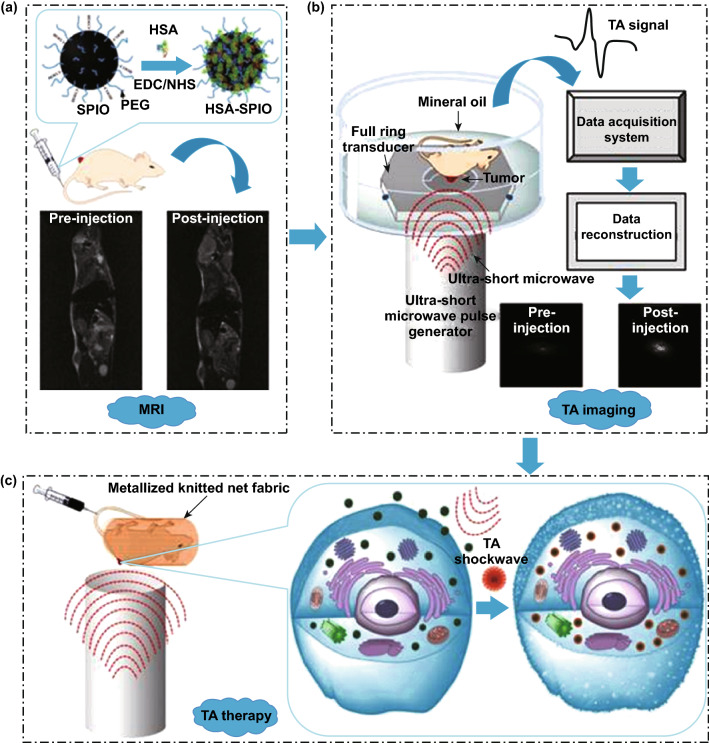
Schematic illustrations of **a** the process of human serum albumin-coated iron oxide NPs (HSA-SPIO) synthesis and their administration to mice, **b** thermoacoustic (TA) imaging system, and **c** HSA-SPIO as therapeutic agent in synergy with MWs. Reprinted under a Creative Common Licence CC-BY 4.0. Copyright 2020 from Ref. [94]

Magnetic NPs have demonstrated to possess good MW absorbing properties and to convert efficiently the MW energy in heat. In particular, iron oxide NPs, specifically with a spherical morphology already known for their capability to act synergistically with MFs or radiation therapy, have demonstrated to efficiently amplify the MW-related toxic effects [21]. Wen et al. proposed to employ human serum albumin (HSA)-coated iron oxide NPs as agent for microwave-pulse-induced thermoacoustic (TA) effect. This is a technique based on the collection of shock waves produced by the rapid thermal expansions of the nanomaterial for imaging purposes. Additionally, the behavior of these NPs could be remotely controlled by magnetic resonance imaging (MRI). Furthermore, these waves are able to perturb the cell membrane integrity, resulting in an augmented cell death, as shown in Fig. 6. In this way, the proposed iron oxide NPs under a MW irradiation could act both as therapeutic tool and imaging agent [94].

Iron oxide NPs could be coated or combined with other nanomaterials to form hybrid NPs with enhanced heating properties under a MW stimulation. An example of coating is tungsten trioxide (WO_3−*x*_). Peng et al. use WO_3−*x*_ as coating agent of iron oxide NPs, demonstrating the improved MW responding properties of these NPs, that are also susceptible to a magnetic stimulation, because of their iron oxide core, enlarging their application as anticancer therapeutics in combination with one of these stimulations or both [97].

It was additionally observed the release of energy from gold NPs subjected to a MW irradiation. These NPs could be employed to improve the efficacy of MW thermal therapy. However, the mechanism has still not been clarified and it is believed to lie on both the temperature increase and ROS production [98]. The synergy between gold NPs and MW is frequently cited to treat the gout disease in the so-called metal-assisted and microwave-accelerated decrystallinization [99, 100]. For anticancer purposes instead, gold NPs and MWs are frequently employed together to maximize the effects of chemotherapy in a combined therapeutic approach, even if in some case the combined action of NPs and MWs is sufficient to induce cell death [101, 102].

Also carbon-based nanomaterials have demonstrated a good synergy with MWs. The nanotubes indeed absorb MW and promote a Joule heating effect [92]. Additionally, also carbon nanotubes are able to produce shock waves under a MW stimulation, thus physically altering cell membranes and organelles provoking cell death. Wen et al. tested single-wall carbon nanotube with a MW treatment as antitumor agent in vitro and in vivo. The nanotubes indeed accumulate in the mitochondria which, following the MW irradiation, produce shock waves. This effect causes injuries to the mitochondria and then apoptosis, resulting in a decrease in tumor volume and an improved survival in mouse models [93]. Beckler confirmed the efficiency of carbon nanotubes with MW for anticancer therapy with multi-walled carbon nanotubes decorated with antiCD44 antibody to target prostate cancer [92].

A completely different approach is based on the use of NPs and MW to achieve cell death through ROS production in the so-called microdynamic therapy. Titania NPs were employed for this purpose on osteosarcoma UMR-106 cells and UMR-106-bearing mice by Chu et al. It was highlighted that only the combination of titania NPs and MW was able to induce cancer cells to apoptosis and to reduce the tumor size. Furthermore, the authors demonstrated that the addition of the NPs did not increase the temperature, suggesting that the observed cytotoxicity was related to another mechanism, lying in ROS production. According to the authors, the MW stimulation generated a plasma in the microbubbles of the solution, with the release of UV light able to induce the formation of free radicals from the NPs. The presence of the NPs also increased the number of microbubbles that could respond to the MW stimulation producing UV light [103]. Tang et al. otherwise observed singlet oxygen production from Cu_2_ZnSnS_4_ NPs under MW irradiation, and they exploited this phenomenon to achieve cell death in both in vitro and in vivo studies [96].

Another possibility is to employ hollow NPs filled by molecules able to enhance the thermal effects of MW. In this case, the coating with NPs has the role to protect the MW enhancer and to deliver it specifically to the target area. Apart from the saline solutions, ionic liquids have been proposed for this purpose since they are characterized by high polarizability [91]. More severe injuries for cancer cells were also reported when NPs are employed to deliver chemotherapeutic drugs, i.e., vinorelbine or doxorubicin [104, 105]. Furthermore, NPs could carry both ionic liquids and chemotherapy drugs to maximize the toxic effects of the MWs [106].

## Light Responding NPs

### Photothermal Therapy

**Fig. 7:**
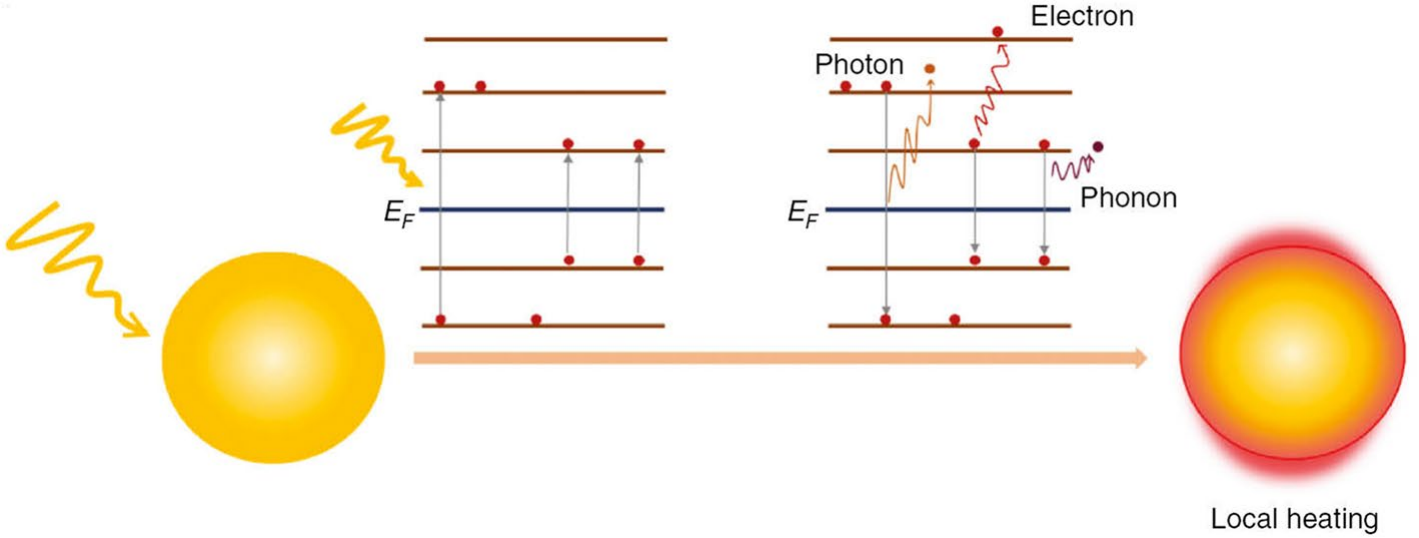
PTT mechanism. Plasmon decay (electron-to-photon, electron-to-electron, and electron-to-phonon) generates local heating. Reprinted under a Creative Common Licence CC-BY 4.0. Copyright 2020 from Ref. [108]

Photothermal therapy (PTT) is a therapeutic approach consisting in the light irradiation and consequent heating of a target region, actually a tumor, over 41 °C, to achieve cell death [107]. This external stimulation could be easily controlled and focused [23]. Light in the near-infrared region (NIR) is usually employed for PTT because of its high-tissue penetration [51]. To maximize the therapeutic outcomes and minimize the thermal injuries to the surrounding healthy tissues, the use of NPs, alone or conjugated with other dyes, capable to increase the temperature after being excited by the NIR light stimulation has been proposed [107]. The mechanism of the conversion of light to heat is believed to lie into the surface plasmon resonance (SPR) effect, but also carbon reticle vibrational state relaxations are sometimes reported [61]. In particular, SPR is caused by the interactions between the light and the electrons of the conduction band of the material that show a coherent motion, termed surface plasmon, upon the irradiation. The excited plasmons can decay in different ways, electron-to-photon, electron-to-electron, and electron-to-phonon, releasing thermal energy, as illustrated in Fig. 7 [108, 109].

Various nanomaterials display high levels of photothermal conversion efficiency [110], but gold is probably the most employed. Depending on the dimensions of the NPs and the medium where they are resuspended, the light could be adsorbed or scattered. Generally, it was demonstrated that the absorption spectra and the plasmon bandwidth are related to the size of the NPs. [23]. Gold nanospheres, nanostars, nanoshells, nanorods, and several other configurations have been tested in PTT [111, 112], and several coatings and/or the conjugation with targeting ligands to achieve a precise focalized action against tumor cells have been evaluated [23]. Very recently, Sancho-Albero et al. proposed to shield PEGylated spherical hollow gold NPs (40 nm diameter) with extracellular vesicles, e.g., exosomes, to evaluate the delivery of such nanotools and their exploitation for PTT. In particular, they decided to incubate different cell lines, healthy and cancer ones, with a non-lethal dose of NPs. After 24 h, they replaced cell culture media with exosomes-free media and 48 h later exosomes containing NPs released from the cells were collected. They reported a highly specific uptake of exosomes-coated NPs depending on the exosome origin, because there is a sort of fingerprint according to their cellular origin. Additionally, such nanotools were able to act in synergy with a NIR light stimulation to decrease cell viability [113].

The same phenomenon is reported for other metals, such as silver, platinum, copper, palladium [22, 51, 53], iron oxide, quantum dots and rare-earth ion-doped photoluminescent NPs [53, 61, 114].

Iron oxide could be employed to form hybrid NPs with other metals, such as gold. These NPs thus have an iron oxide core and a gold shell, exploiting in this way the magnetic properties of iron oxide for imaging through MRI, whereas the gold shell, upon a NIR irradiation, causes a temperature increase and cell death. These hybrid NPs are even useful for combined therapeutic approaches involving more than one physical stimulation to maximize the therapeutic outcomes. They could also be coupled with other metals, or employed for the delivery of drugs or other PTT enhancer molecules [114].

Since the abovementioned NPs present some limitations to the translation to the clinic, such as the low biodegradability, several other nanomaterials have been proposed as PTT-enhancers, starting from carbon-based ones [53]. In particular, single- and multiple-walled carbon nanotubes and graphene oxide NPs are good light-responsive nanostructures already employed in PTT investigations. Several example could be found in the literature [23, 51, 53, 61]. The carbon nanostructure indeed is able to absorb light and convert into vibrations of the lattice, releasing energy in the decay [61]. An example could be represented by the study of Lu and co-workers. These authors targeted pancreatic tumors with PEGylated single-walled carbon nanotubes conjugated with the NIR fluorescent dye Cy7, to track nanotube internalization, and the anti-insulin like growth factor receptor type 1 toward pancreatic cancer cells. They observed a marked reduction in viability only in cells incubated with the nanotool and treated with NIR light. Monitoring the ROS production, it was furthermore evidenced a considerable improvement in the ROS level in the same cells. In addition, with orthotopic pancreatic cancer-bearing mice they confirmed the efficient internalization of the proposed nanotool an augmented heating in the tumor area after the NIR irradiation [115].

Some nanomaterials are capable to absorb light at other wavelengths, and thus they are less suitable for PTT applications. However, several researches tried to tune their absorption for PTT purposes. An example is represented by titania NPs. As reported by Zhang et al., titania is normally characterized by an intense UV absorption and is employed for the photodynamic therapy. However, chemical modifications can tailor its absorption to NIR, allowing its use as PTT agent. Oxygen‐deficient black titania (TiO_2−*x*_) NPs, obtained starting from Al reduction in titania, or Nb-doped titania NPs have both demonstrated to efficiently convert NIR absorption to heat, inducing cancer cells to death [19]. Mou et al. administered PEGylated TiO_2−*x*_ NPs to tumor-bearing mice. After their accumulation in the tumor, NIR light irradiation was performed, causing the increase in the temperature and tumor ablation. Furthermore, it was highlighted the capability of these NPs to convert light energy also into chemical energy, generating ROS, obtaining a sort of combined photodynamic-photothermal therapy [116].

Several polymeric NPs have also been successfully employed as anticancer agent in PTT. Indeed, polymers as polypyrrole, poly-(3,4-ethylenedioxythiophene):poly(4-styrenesulfonate), dopamine-melanin, and polyaniline have been used to build biocompatible and light-responsive NPs to target cancer cells alone or combined with other molecules [51, 117]. A remarkable example of polymeric NPs for PTT is represented by the investigation of Wang et al. The authors synthetized a hybrid system composed by lipids and polyaniline, decorated with folic acid, to improve the tumor targeting. Such NPs demonstrated useful properties exploitable for both imaging and PTT in HeLa cells and BALB/c mice-bearing HeLa tumor [118].

Another possibility is to conjugate NPs already employed in PTT with chemotherapeutic agents, or immunoadjuvant drugs, as revised elsewhere [107, 117].

### Photodynamic Therapy

**Fig. 8:**
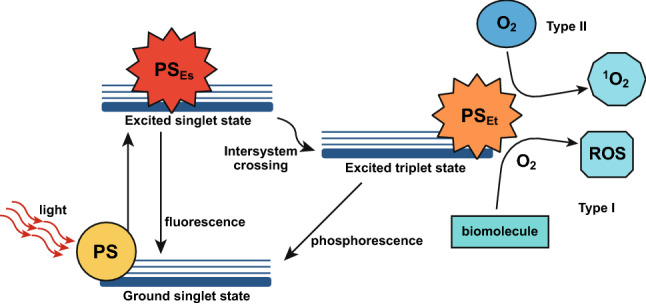
Jablonski’s diagram illustrating PDT mechanism. The photosensitizer (PS) is excited from a ground state to an excited single (PS_Es_) and then a triple (PS_Et_) state, in which it could react in two ways (Type I and II). Reprinted under a Creative Common Licence CC-BY 4.0. Copyright 2020 from Ref. [138]

Another possible combination of light and NPs to achieve cancer cell death is the photodynamic therapy (PDT). This treatment consists in the administration of a molecule, named photosensitizer, that is excited by a light with a determinate wavelength, resulting in the generation of different ROS species able to cause cell death [119]. After the light excitation, the photosensitizer passes from a ground state to an excited single and then triplet state, where it can directly react with several biomolecules, as lipids and proteins, forming different radicals (Type I reaction). Alternatively, it can react with molecular oxygen, with singlet oxygen (^1^O_2_) production (Type II reaction), as shown in Fig. 8 [119, 120].

ROS and more in general free radicals are responsible of the generation of several injuries to the cells, inducing apoptosis, necrosis or autophagia depending on the intracytoplasmic localization of the photosensitizer, the energy applied, and other factors [119]. ROS and free radicals can moreover impair the tumor vasculature, enhancing inflammatory responses and then activating an antitumor immune system-specific reaction [121]. Several classes of photosensitizers have been approved for clinical use, as recently reported elsewhere [119, 122]. Conventional photosensitizers are porphyrins, chlorins, and other molecules, as chlorophylls. They all have to face problems as the administration into the human body, the tumor selectivity, the degradation, and the photobleaching [121]. For these reasons, several NPs have been proposed alone or combined with a photosensitizer to improve the efficacy of PDT [123–125].

Some nanomaterials indeed, actually carbon [126], zinc oxide, and titanium dioxide [127, 128], have demonstrated to be able to generate ROS after a light stimulation, acting as photosensitizers themselves. Fullerenes, composed by 60 or 70 carbon atoms in a spherical shape, are capable to absorb light in the UV or blue region, generating both free radicals and singlet oxygen. They have been extensively employed as photosensitizers in PDT because they conjugate good photosensitizing properties with a high photostability and a low susceptibility to photobleaching. Since they are insoluble in water, they are usually proposed with surface modifications and/or conjugated with coating agents for biological applications. Furthermore, they can be conjugated with other molecules for imaging, creating theranostic NPs [125]. Grebinyk et al. evaluated the accumulation and localization inside human leukemic cells of C_60_ fullerene NPs and their photosensitizing properties induced by UV, violet, green, and red high-power single-chip LEDs light irradiation. The results evidenced the NPs localization in mitochondria of human leukemic cells. The highest cytotoxicity was recorded when NPs where combined with a UV irradiation, whereas no toxic effects were reported when the NPs were associated with an irradiation of green or red light, because of the lower absorption of the NPs in these regions. Additionally, the UV irradiation resulted in an increased ROS production and apoptosis [129].

**Fig. 9:**
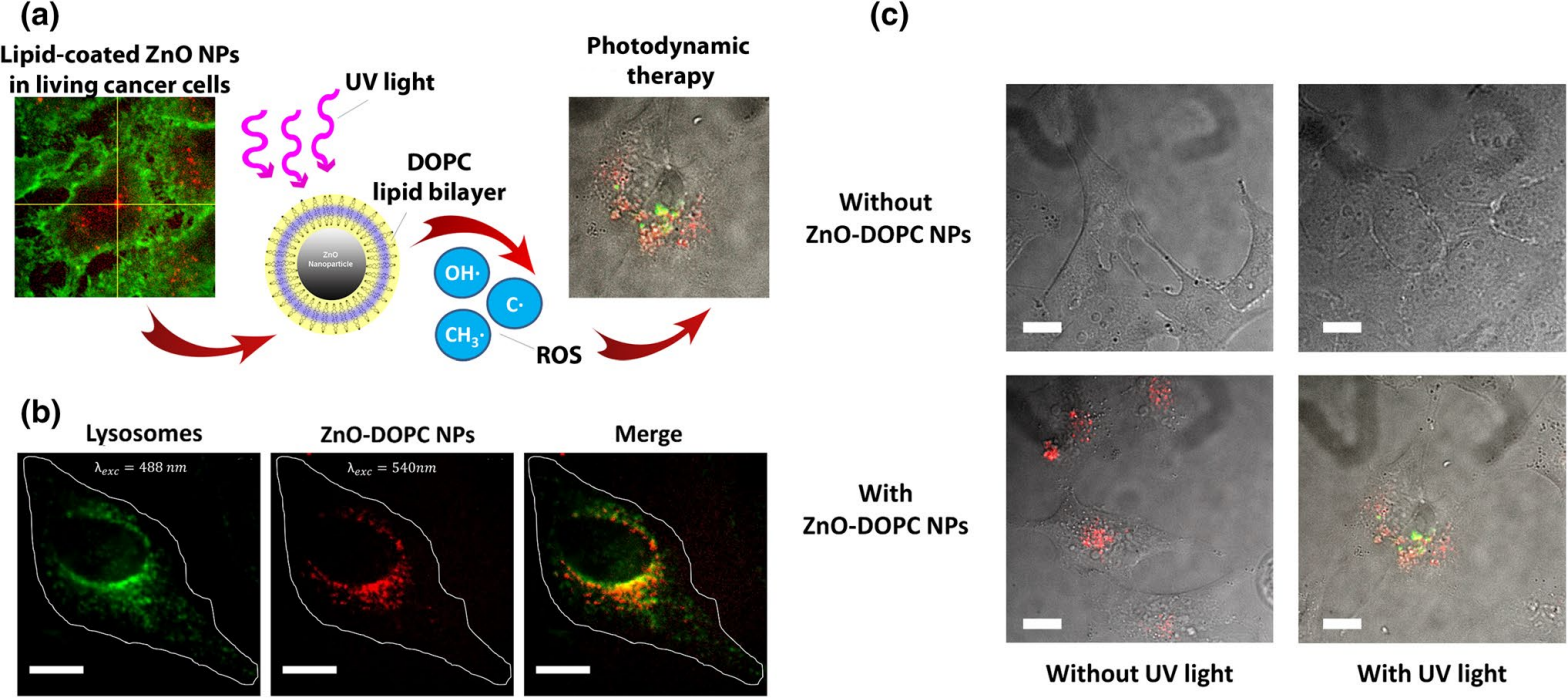
**a** Schematic representation of PDT with lipid coated zinc oxide NPs (ZnO-DOPC NPs). **b** Fluorescent-labeled (Atto550-NHS) ZnO-DOPC NPs colocalization with lysosomes labeled with Lysosomes-GFP. **c** ROS generation measurement through the 2′–7′dichlorofluorescein diacetate assay. Scale bars: 5 μm. Reprinted under a Creative Common Licence CC-BY 4.0. Copyright 2020 from Ref. [130]

It was demonstrated that the semiconducting zinc oxide NPs exposed to an UV stimulation are able to generate ROS. The irradiation with a light characterized by an energy higher than their band gap (3.3 eV) indeed induces an electron transfer from the valence band to the conduction band, with the creation of an electron–hole pair. The electrons can reduce oxygen molecules thus forming superoxide radical anion, while the holes can oxidize water molecules and hydroxide ions, generating hydroxyl radicals and hydrogen peroxide [10]. In this context, our group demonstrated through electron paramagnetic resonance studies that pristine zinc oxide NPs (ZnO NPs) were not able to induce ROS generation without an external stimulation. However, after the irradiation under UV light, NPs produced an impressive increase in ROS, in particular hydroxyl radicals, capable to exert cytotoxic effects in HeLa cells. This phenomenon was not affected by the presence of a phospholipidic bilayer around the NPs, composed by 1,2-dioleoyl-sn-glycero-3-phosphocholine (DOPC) to allow their better dispersion in biological fluid and higher cell internalization, suggesting that the presence of the coating agent does not interfere with the pathway of ROS generation. As depicted in Fig. 9c regarding the ROS generation of HeLa cells W/O UV stimulation and W/O lipid-coated ZnO NPs (ZnO-DOPC NPs), it was highlighted that only in the presence of ZnO-DOPC NPs and UV light there was ROS production, responsible of the observed cell death reported with the MTT assay [130].

Titania, another semiconductor material characterized by a band gap energy comparable to zinc oxide, possesses almost the same light-responsive properties, and additionally, it is associated with a very low toxicity [19, 125]. Several examples of titania NPs have been thus proposed for PDT [19, 131, 132]. A peculiar implementation through a reduction in conventional titania is the black titania. This reaction induces the formation of Ti^3+^ ions on the surface of the NPs and modifies their photosensitizing properties, shifting their absorption from UV to NIR. Ni et al. observed that this peculiar modification allowed the NPs to act in synergy with NIR light causing bladder cancer cell death [133].

Sometimes, two or more materials are coupled to form hybrid NPs with enhanced light responses. Gold, copper, iron and other materials have been proposed in combination with zinc oxide for this purpose, because they tune its absorption from UV to visible light or NIR, that are characterized by a higher tissue penetration [134]. When zinc oxide is combined with copper, e.g., and irradiated with visible light, it was observed that a reaction occurs between the electrons present in the valence band of zinc oxide and the copper, with the conversion of Cu^2+^ to Cu^+^. This last generated species and the positive holes on the NPs surface both react with oxygen, hydroxyl groups and water with a consequent ROS formation [135]. Another possibility is the doping of the NPs with elements that are able to absorb in the NIR light range or the X-rays. These radiations indeed possess a deeper tissue penetration, in contrast with the UV generally employed to induce the production of ROS [134]. In the case of NIR, this is achieved conjugating the NPs responsive to PDT, i.e., titania and zinc oxide NPs, with the so-called up-conversion NPs, that are usually comprised of host lattices of ceramic materials, embedded with transition metal, actinide or lanthanide ions and are able to absorb NIR light and excite the NPs releasing visible light [125]. Otherwise NPs could be conjugated with nanoscintillators that possess the capability to convert gamma-rays or X-ray onto a visible light. The resulting therapeutic approach, also called X-ray PDT [136], consists on the conjugation of the photosensitizer to a nanoscintillator, the internalization of such nanotool by cancer cells and the administration of a radiation treatment [137]. Several nanomaterials have been developed for this particular PDT, as metal–(in)organic clusters, metal materials, radioluminescent nanophosphors, and quantum dots, as precisely reported in a recent review [136].

NPs could be also employed to deliver the photosensitizers. Several types of inorganic NPs, e.g., silicon and gold ones, as well as liposomal and polymeric biodegradable systems, have been proposed as nanocarriers for the delivery of the photosensitizers with promising results, as revised elsewhere [125, 138, 139]. Additionally, the nanocarrier could contain not only the photosensitizer, but also an imaging agent, becoming then a theranostic tool [139]. Yang et al. adopted hollow manganese dioxide nanoplatforms, which ions improve MRI contrast, as carrier of both the photosensitizer chlorine e6 and anticancer drug doxorubicin for a combined chemotherapy-PDT [140].

## Mechanical Wave-Based Therapies

### Ultrasound-Responsive NPs

Ultrasound (US) is a mechanical sound wave with a periodic vibration frequency higher than human hearing [141]. The irradiation with US causes both thermal and non-thermal effects. Thermal effects derive from the passage of the US wave in the tissue, where part of its mechanical energy is dissipated in heat through friction effects, potentially causing hyperthermia and cell death. Otherwise, non-thermal effects depend on the acoustic cavitation and its consequences. This phenomenon is related to rarefaction and compression cycles caused by the US field, in which gas pockets, already present in the body, grow and form microbubbles that expand and shrink. In the so-called stable or non-inertial cavitation, the microbubbles oscillate for several acoustic cycles, heating the irradiated area and causing mechanical stress through the generation of microstreamings, radiation forces, and shear stress. On the contrary, in the inertial cavitation the microbubbles collapse generating very high pressures and temperatures, inducing also the formation of several ROS trough the sonolysis of water molecules or perturbing molecules in close proximity to imploding bubbles. The collapse of the gas bubbles results also in mechanical stress for the cells, with the generation of microjets and shock waves. Additionally, a characteristic emission of light, named sonoluminescence, is reported as a consequence of the microbubble implosion, even if its exact mechanism has not been fully understood. The predominance of thermal or non-thermal effects depends on the parameters of the applied US field [16, 142].

US have been adopted in different fields for diagnosis and therapy, included anticancer therapy. High-intensity US indeed have been employed to drastically improve the temperature in a focal region, actually a tumor, obtaining the complete tumor ablation through coagulative necrosis [141, 142]. In addition, US can be employed alone or in combination with gas bubbles to facilitate the internalization of drugs or nucleic acids temporarily altering the permeability of plasma membranes [16].

In the last years however, several researchers, inspired by the promising results of PDT, proposed to couple the US input with photosensitizer molecules at the beginning, and then NPs to maximize the therapeutic outcomes. This approach is called sonodynamic therapy (SDT). The applications of US in combination to a molecule, called in this case sonosentizer, are object of numerous research papers and reviews [16, 143, 144]. Different NPs could be employed to deliver the sonosensitizer in a desired site preserving its properties in the biological environment [143]. Moreover, in the last years, several groups proposed the use of the sole NPs as sonosensitizers [16, 144].

**Fig. 10:**
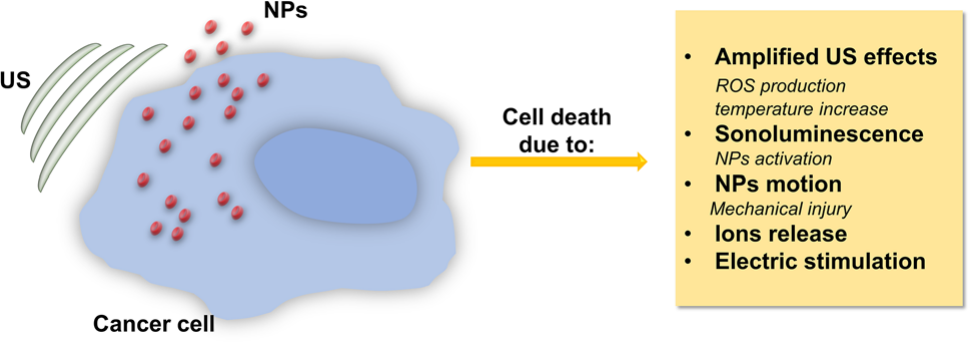
Summary of possible mechanisms to explain the cytotoxicity arising from NP-assisted US therapy

The exact mechanism of this synergism has not been fully understood yet; however, a possible explanation is shown in Fig. 10. It is worth to mention that the mechanism of action depends obviously on the features of the nanomaterial involved and on US parameters.

During the NPs-assisted US therapy, both effects arising from the US irradiation alone, thermal and non-thermal ones, and the combination of US with the NPs have to be considered.

The addition of the NPs decreases the cavitation threshold that is US dose necessary to obtain acoustic cavitation. It was experimentally demonstrated that NPs act as nucleation site thanks to their surface roughness or the presence of pores in their structures that allow to carry tiny gas bubbles. These gas nuclei can form bubbles that grow and collapse or persist for many acoustic cycles, amplifying the effects of the US [145].

Otherwise, sonoluminescence-derived light radiation could be able to activate light-responsive NPs (e.g., gold or semiconductor NPs), with different consequences (e.g., temperature increase), ROS production and others, dependent on the material involved [16].

Another possible mechanism about the combination between US and NPs was proposed by Osminkina et al. It lies on the mechanical stress provoked by the NPs motion inside cancer cells under a US stimulation, that the authors identify as “nanoscalpel effect”, that could be able to cause mechanical injuries that induce cell death [146].

For specific chemically unstable NPs (e.g., zinc oxide and iron oxide ones), the US stimulation could also provoke the NPs disaggregation and enhance their degradation with the release of toxic ions, resulting in cell death [147, 148].

In the case of NPs made by piezoelectric materials (e.g., barium titanate), the US stimulation could generate electric charges that impair cell functions [149].

Since there are several possible modalities in which US can cause cell death in synergy with NPs, a very large number of different nanomaterials have been proposed as sonosensitizers [16, 143, 150].

Gold [151, 152] and silver [153] NPs were both proposed as sonosensitizers. Brazzale et al. developed folate-decorated gold NPs and tested their properties for SDT purposes in different cancer cell lines. They chose gold because of its peculiar optical properties and decided to functionalize these NPs with folate because many tumors overexpress the folate receptor. These NPs are indeed able to decrease the cavitation threshold, but could also increase the temperature through a SPR-related mechanism, probably due to a sonoluminescent excitation of the NPs. Furthermore, the warming could supply more gas for the formation of the bubbles under a cavitating regime. They successfully demonstrated the selective uptake of folate-decorated NPs and that their synergy with US was able to significantly increase ROS production and decrease cell viability [152].

However, titania-based NPs are probably the most investigated nanotool for SDT applications. In fact, as gold NPs, they provide cavitation nuclei and could be excited by sonoluminescence, improving ROS production and cell death. Several investigations have thus been carried out in this direction [19, 154]. Titania NPs are often functionalized with PEG and/or other molecules to prevent aggregation and increase their therapeutic effects. Additionally, they could be synthetized with a mesoporous conformation to entrap drugs or combined with other materials to form hybrid NPs with enhanced anticancer properties. Gold-coated titania nanoparticles were developed to increase sonochemical reactions. In fact, the presence of gold is able to trap the sono-excited electrons and reduce electron-holes fast recombination, enhancing ROS production [19].

Zinc oxide moreover, presenting very similar chemical properties, is a promising candidate for SDT. Our group recently investigated the ability of amino-propyl functionalized zinc oxide nanocrystals to induce inertial cavitation after pulsed US exposure, observing a large production of ROS, specifically of hydroxyl and superoxide anions. The adopted US conditions were thus sufficient to initiate the acoustic cavitation of tiny gas bubbles trapped at the surface of the nanocrystals [31].

Also, magnetic NPs could be used for SDT [148, 155, 156]. Ebrahimi et al. investigated the sonodynamic properties of iron oxide nanoparticles in a breast carcinoma cell line. They showed an increased cytotoxic effect when they improved the concentration of NPs subjected to a US exposure. This was perhaps due to iron ions release, because they can react with hydrogen peroxide, forming hydroxyl and hydroperoxyl radicals through Fenton reaction, increasing ROS burst and cell death [148].

Gong et al. otherwise suggested the use of oxygen-deficient MnWO_X_ NPs as sonosensitizers. With these NPs indeed a highly effective SDT treatment of tumors was achieved. The oxygen-deficient structure could provide electron trapping sites to prevent electron–hole recombination, causing the production of a large amount of ROS. Additionally, NPs showed a unique capability of glutathione depletion, which increases SDT-triggered cancer cell killing [157].

Silicon has been employed to build active NPs under US. Osminkina et al. [146] synthetized dextran-coated silicon NPs and investigated their potential application as sonosensitizer in vitro and in vivo. The observed enhanced cell death was explained with SDT-associated hyperthermia and mechanical damages caused by the internal motion of the NPs that results in apoptosis, actually the previously mentioned “nanoscalpel effect”. Furthermore, investigating the interactions between silicon NPs and US, Sviridov et al. highlighted two main mechanisms. (1) an enhanced scattering and viscous dissipation of the US energy in the medium with NPs, resulting in the heating of the medium; (2) an augmented acoustic cavitation-associated heating, more pronounced in the case of NPs with hydrophobic inner walls [158].

The use of carbon-based nanomaterials as sonosensitizers is also reported in the literature [159, 160]. In this context, Kharin and group proposed a new theranostic agent consisting of fluorescent carbon NPs. They observed the preferential accumulation of these NPs in both the nuclei of healthy and cancer cells after the internalization. Furthermore, the subsequent treatment with US resulted in a mechanical injury and cell death for both the cell lines considered, highlighting the importance of the tumor targeting [159].

Another possibility is to employ materials with piezoelectric properties to electrically stimulate cancer cells exposed to US [149, 161]. For this purpose, Marino et al. used biocompatible piezoelectric barium titanate NPs functionalized with epidermal growth factor in order to target breast cancer cells. US treatments were performed 1 h once a day for 4 days to cells W/O NPs. Later, they evaluated cell metabolism, cell cycle, and the morphology of the mitotic spindles. They recorded a drop of the metabolic activity, a stop of cell proliferation, and mitotic aberrations in cells treated with both nanoparticles and US, confirming the existence of a synergistic effect [149]. The same strategy was adopted against glioblastoma multiforme in a second paper [161].

### Shock Wave-Responsive NPs

**Fig. 11:**
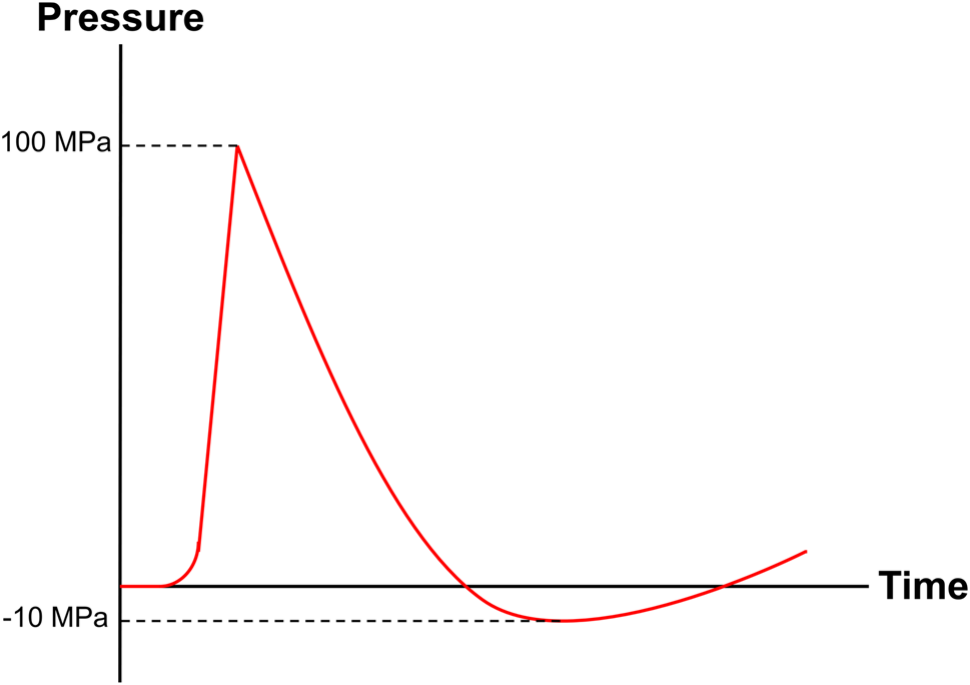
Scheme of a therapeutic SW

Shock waves (SW) are mechanical waves characterized by a first very high peak pressure (up to 100 MPa) with a phase duration of 0.5–3 μs, followed by a tensile wave characterized by a negative pressure (− 10 MPa) for 2–20 s, before recovering to ambient values [144]. Figure 11 shows the typical form of a therapeutic SW.

The extracorporeal SW therapy is nowadays clinically employed for the treatment of several diseases characterized by different origin and affecting different organs, such as tendon-related pathologies, wound healing, and others. Part of the SW therapeutic outcomes lies on the so-called mechanotransduction effect. This is composed by the molecular mechanisms that regulate the responses at the mechanical stimulations, and influences processes such as migration, proliferation, differentiation, and apoptosis [162]. SW have also been proposed alone to inhibit the growth of cancer cells. Foglietta et al. reported that when mesenchymal stem cells and cancer cells (glioblastoma or osteosarcoma) co-cultures are irradiated with SW, the consequence is a selective cancer cell death related to the ROS production by mesenchymal stem cells [163]. Otherwise, SW could be employed to permeabilize temporarily cell membrane and improve the uptake of drugs and chemotherapies [164, 165], as well for the delivery of nucleic acids [166, 167].

Additionally, some researchers proposed to use SW in SDT investigations. This proposal was made because, despite the promising anticancer results obtained with the SDT in different cell lines and tumor models, an important limitation is represented by the understanding of its operating principle, even if it is believed that the main mechanism lies in the inertial cavitation. The use of high-energy SW to activate the sonosensitizer allows to minimize the thermal effects related to US and to precisely observe the non-thermal ones and their consequences [144]. Some authors thus employed SW in synergy with canonical photosensitizers in order to achieve cancer cell death [168–170]. In this case, NPs could be exploited to deliver the sonosensitizer and improve its internalization in cancer cells [171, 172]. Varchi et al. synthetized poly-methyl methacrylate NPs (PMMANPs) carrying the meso-tetrakis (4-sulfonatophenyl) porphyrin (TPPS), observing an enhancement of the sonosensitizing action of the TPPS when loaded into the PMMANPs in terms of reduction in tumor volume in a breast cancer model, and additionally recorded an improved expression of genes related to oxidative stress [171].

**Fig. 12:**
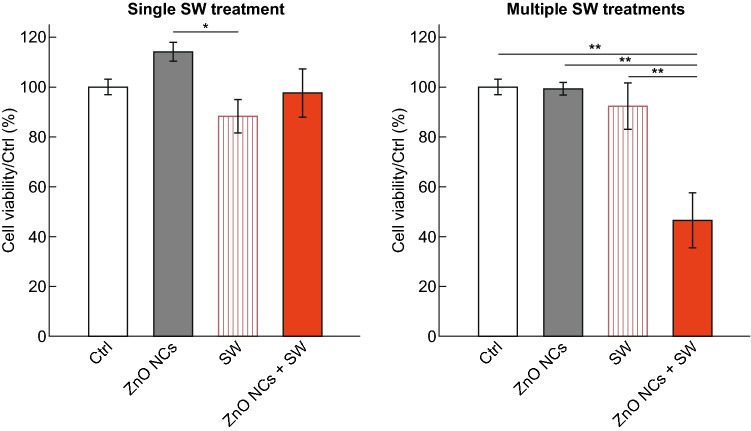
Comparison between single toward multiple SW treatments on cancer cells co-incubated with ZnO NCs. Samples: untreated cells (Ctrl), cells incubated with ZnO NCs (ZnO NCs), cells treated with SW (SW), cells incubated with ZnO NCs and treated with SW (ZnO NCs + SW). Results are shown as mean ± SEM. *p < 0.05, **p < 0.01. Adapted from Ref. [25]

Some NPs could be also employed as sonosensitizers to study the synergy with SW, but from our knowledge, only our recent study with zinc oxide nanocrystals (ZnO NCs) and SW with different positive peak pressure (PPP) successfully conjugated NPs with SW. We indeed compared the effects of a single toward multiple SW stimulations in cervical adenocarcinoma cells co-incubated with ZnO NCs. We discovered that only multiple stimulations (three treatments/day) were able to trigger the synergy with ZnO NCs and thus induced cancer cells to death (Fig. 12). The observed cell death is probably related to a multifaceted mechanism potentially involving some of the pathways proposed above, as the amplification of US effects, a partial NPs dissolution with the release of Zn^2+^ ions, which imbalance is responsible of different alterations in cell behavior, and perhaps to a situation of mechanical stress caused by the motion of the internalized NPs under the SW stimulation [25].

## Combined Stimuli for Enhanced Therapy

Generally, the NPs are triggered by a single remote stimulation, but there are some examples in the literature of NPs activated by the simultaneous administration of two or more physical inputs, maximizing cancer cell death. There are nanomaterials responsive to more stimulations, as gold [173]. Otherwise, hybrid NPs comprising materials with different properties could be employed, and each component could interact with a single input [83]. This has indeed the remarkable advantage to increase the therapeutic outcomes further reducing side effects [174], and several possible strategies are reported in the literature.

A first possibility is to employ nanomaterials with the intriguing property to improve the radiation therapy efficacy and also able to generate hyperthermia in association with a second stimulus. Hyperthermia indeed is able to radiosensitize cancer cells inhibiting DNA repair and, at the same time, increasing the oxygenation of the tissues acting on the blood flow. In this way, the radiation dose required to obtain the complete tumor ablation is reduced, limiting thus the side effects associated to this therapeutic approach [175]. MF-responsive NPs could be employed for this purpose, and furthermore, they could be magnetically delivered to a target area and used for imaging. Magnetic NPs, as iron oxide ones, and hybrid NPs have been proposed in this context [40]. Anyway, in this particular combination usually only one of the stimulations activates the NPs to trigger cytotoxicity. In this example, Jiang et al. adopted gadolinium-doped iron oxide NPs that in synergy with a magnetic stimulation generate both thermal ablation and mild hyperthermia in a mouse model. This effect improved the efficacy of the subsequent radiation therapy through the reduction in the radiation resistance, decreasing the fraction of hypoxic cells, and perturbing the tumor vasculature [176]. In another case on the contrary hybrid NPs acted in synergism with the radiation therapy, while the magnetic stimulation was employed only for imaging purposes [177].

Otherwise, the combination of radiation therapy with PTT has been widely explored. A first possibility is to employ a multi-responsive nanomaterial, as gold. Gold nanostructures indeed generate heat when excited by light and additionally their radiosensitizing effect is reported in the literature as abovementioned. Various morphologies of gold nanostructures have been therefore employed for a double stimulation with light and radiation therapy [178, 179]. More in general, several metal NPs could potentially be involved in the same exploration, as bismuth [180] and platinum NPs, which anticancer efficacy in combination with radiation therapy and PTT has been recently demonstrated [181]. Also hybrid NPs, such as copper sulfide [182] or composed by gold and iron oxide, have been largely employed in the combined PTT-radiation therapy approach. Furthermore, some of these nanohybrids could be potentially activated also by a magnetic stimulation, opening the possibility to insert a third physical input, even if for imaging purposes [183]. In this context, Mohavedi et al. recently deepened the mechanism of this synergy, demonstrating that gold-coated iron oxide core–shell NPs are accumulated in mitochondria after the internalization in KB cancer cells. The double stimulation by light and radiation therapy causes a massive cell injury, observable through transmission electron microscopy, and activates the expression of apoptotic genes. Also the overexpression of the HSP70 gene, associated to cell heating, inflammation, radiation and others stress stimuli, has been recorded [184].

Considering instead the possibility to combine radiation therapy with PDT, another opportunity is to conjugate a photosensitizer with a radiation-responding material [185]. From the best of our knowledge, however, only conventional photosensitizers have been involved in these studies. NPs indeed are sometimes employed merely as carrier, without playing an active role in synergy with the stimulations [138, 185].

Radiation therapy is often associated to US for imaging purposes [186], and furthermore US irradiation alone can radiosensitize cancer cells [187]. Shanei et al. moreover proposed to add gold NPs that are able to interact synergistically with both X-ray and US triggering cancer cell death. The authors indeed measured the sono-radiosensitivity effect of different concentrations of gold NPs on HeLa cells, tuning the parameters of both US and X-rays. They found that all the considered concentrations improved cell mortality when remotely activated by the two inputs [174].

Leaving the radiation therapy, there are moreover some interesting proposals where nanomaterials are remotely activated by a MF and another physical stimulation. It is reported in the literature that the combination of magnetic hyperthermia and PTT has a tremendous impact on cancer cells, because their interaction is very effective, overcoming furthermore the limitations and the drawbacks associated with the single magnetic hyperthermia or PTT [188]. In this context, some studies with NPs have been presented, involving both NPs made by a single material and hybrid NPs in which each component is remotely activated by a different input, even if in some cases the magnetic stimulation has only imaging purpose [61, 62]. Espinosa et al. demonstrated that iron oxide nanocubes remotely triggered by MF and a light input provoked a more marked temperature increase than when the nanocubes were irradiated by only one of the stimulations. This synergistic or cumulative effect allowed the complete cancer cell death in vitro and tumor ablation in vivo [189]. Ma et al. additionally proposed the use of hybrid NPs formed by iron oxide and palladium. These NPs indeed possess magnetic properties, due to the iron oxide component, the capability to generate ROS in the presence of hydrogen peroxide through to Fenton reactions, and PTT responsiveness due to the palladium. Actually, palladium possesses a strong SPR band in the NIR region and moreover could generate ROS in acidic conditions. The authors recorded a high-temperature increase with the double-activation of the NPs, and additionally an improved ROS generation [190].

PDT has also been proposed in combination with a magnetic stimulation. The aim of the MF input could be also in this case for imaging and for the magnetic guidance of the NPs; however, sometimes MF can also contribute to cell death. The nanosystem involved in this case is generally composed by NPs with magnetic properties conjugated with a photosensitizer, maximizing the therapeutic outcomes under a double magnetic-light stimulation [188, 191]. Curcio et al. synthetized hybrid NPs constituted by a nanoflower-like iron oxide structure and a copper sulfide shell. The iron oxide core indeed increased the temperature under a MF, while the copper shell made them responsive to light for both PTT and PDT [192].

Magnetic hyperthermia could be also combined with US, and there are some examples in the literature of this dual-stimulation. Jozefczak et al. demonstrated that the temperature of a tissue mimicking phantom showed a more marked increase when iron oxide NPs were irradiated by both MF and US, rather than the single stimulation. This effect was explained as the NPs improved the absorption of US energy and were also capable to produce heating under the MF [193]. Despite of this, the MF role is often limited to imaging and targeting when combined with US [157, 194].

In contrast with all the modalities for a remote triggering of the NPs presented above, MW have been very rarely involved in a dual-stimulation mode of NPs, and from our knowledge, no cases of MW-PTT and MW-PTT involving NPs as therapeutic agent are reported. US otherwise have been sometimes employed in combination with MW to improve tumor ablation. The localized impairment of tumor vasculature following the acoustic cavitation indeed could improve the efficacy of the MW-associated hyperthermia decreasing the heat loss [195]. Otherwise, US could be involved for imaging purposes, e.g., to monitor the MW thermal ablation [196], thus potentially the addition of NPs could drastically improve the therapeutic effects. Gebreel et al. demonstrated the enhanced cytotoxicity of iron oxide NPs stimulated by both US and MW in tumor-bearing mice, with a 97.89% reduction in the tumor volume following the dual-mode stimulation [197].

Besides, since both PDT and PTT possess a high therapeutic efficacy and low side effects, several researches have attempted their combination to maximize cancer cell death. In this case, hybrid complexes formed by a PTT-responsive NP and a conventional photosensitizer have been proposed [198, 199], as the use of sole NPs opportunely build to improve both PTT and PDT outcomes. A possibility is to employ NPs synthetized with a single material responsive to both the stimuli, as gold. Actually, gold has the capability to generate ROS under certain conditions of irradiation, perhaps though a plasmon-activated pathway or an indirect modality. Unfortunately, this mechanism is not well explored, as the majority of the studies in the literature presents gold NPs as carrier of photosensitizer [200], instead of the sole gold NPs in synergy with light for a dual PDT–PTT [173]. Also black titania nanostructures could be employed for this purpose [116]. An alternative is to design hybrid NPs with improved photosensitive properties [201]. In this latter case, Lee et al. synthetized a nanocomplex constituted by defective titania NPs that generates ROS when exposed to NIR, and gold nanorods, as PTT-enhancers. When HeLa cells, previously incubated with the nanocomplexes, were subjected to both PDT and PTT, a significantly reduced cell viability was observed and associated to ROS burst, produced by titania NPs, and temperature increase, due to the irradiation of the nanorods [202].

**Fig. 13:**
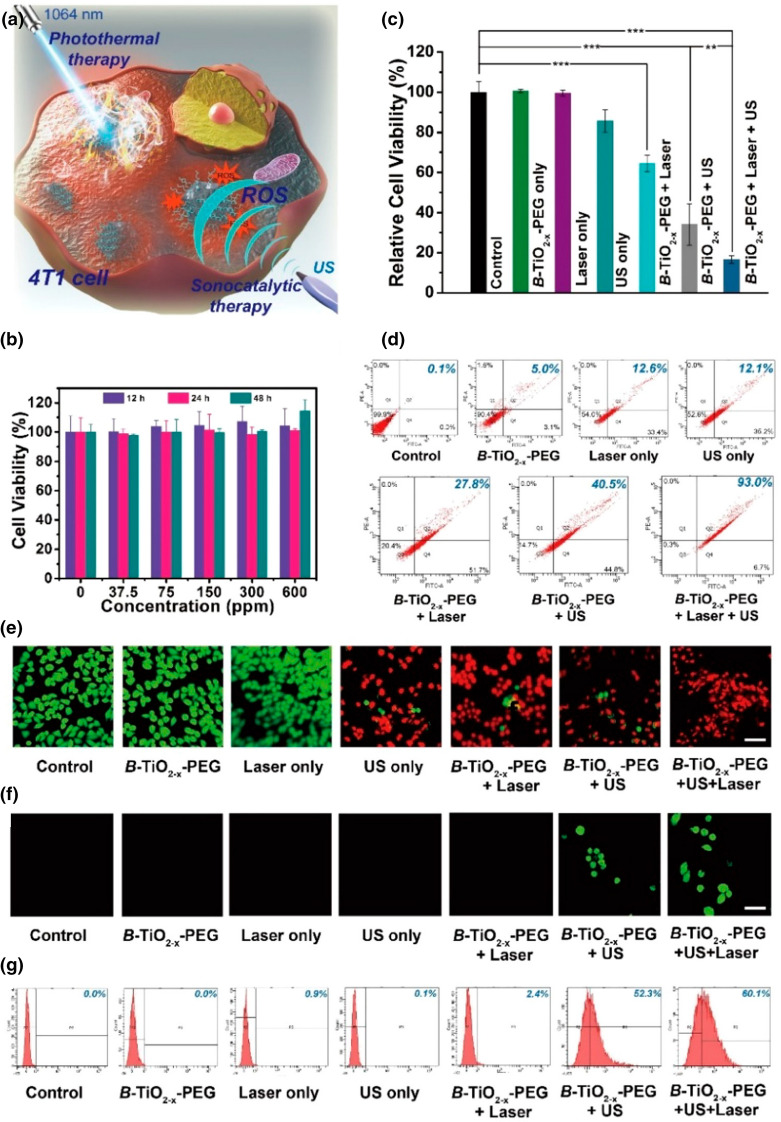
Combined effect of NPs with light and US. **a** Scheme illustrating the synergy proposed. **b** Cell viability after incubation with the proposed nanosystem (B-TiO_2−*x*_-PEG). **c** Cell viability after different treatments. ***p < 0.001. **d** Flow cytometry apoptosis assay after different treatments, staining with Annexin-FITC and PI. **e** Confocal laser scanning microscopy images of after different treatments, PI (red fluorescence), calcein-AM (green fluorescence). Scale bar 40 μm. **f** Confocal laser scanning microscopy images of cells stained with 2′–7′dichlorofluorescein diacetate after different treatments, scale bar 20 μm. **g** Flow cytometry measurement of ROS production. Adapted with permission from [154]. Copyright 2020 American Chemical Society

Moreover, focusing of light-US dual-activated NPs, there are several examples of NPs for a combined SDT–PTT. Sazgarnia et al. proposed gold NPs excited by light and US. In this context, indeed gold NPs could decrease the cavitation threshold delivering gas pockets on their structure and additionally, when irradiated by NIR light, they induce a temperature increase. The temperature rise moreover causes the vaporization of the surrounding medium, providing vapor bubbles which are active under the US field. The administration of the NPs and the irradiation with both inputs resulted to inhibit more efficiently the tumor growth and improves the cumulative survival fraction in colon carcinoma bearing mice [203]. Han et al. exploited otherwise NPs composed by black TiO_2_@TiO_2−*x*_ core/shell nanostructures composed by titania nanocrystals with an oxygen-deficient surface layer for a combined PTT-SDT anticancer therapy. The oxygen deficiency indeed facilitates the electron-holes separations under US, improving the regeneration of ROS, and furthermore this nanosystem is responsive to NIR, triggering hyperthermia. NPs were non-toxic for cancer cells in vitro (Fig. 13b), but only when activated by both laser light and US, as confirmed by the flow cytometry assessment of apoptosis and fluorescence microscopy analysis (Fig. 13c–e, respectively). Furthermore, ROS production resulted to be maximized when the NPs acted synergistically with both the inputs (Fig. 13f). The proposed treatment was additionally able to completely eradicate the tumor in a mouse model [154]. There are besides several examples of hybrid titania NPs for an enhanced PTT-SDT anticancer treatment [204, 205]. Otherwise when PDT is combined with SDT, from the best of our knowledge, one of the components is always a conventional photosensitizer, i.e., a porphyrin, not involving the NPs as sole therapeutic agent [16, 144].

## Clinical Trials with Remotely Activated NPs

NPs have been widely investigated for the treatment of different diseases, in particular for anticancer purposes.

The use of NPs as anticancer agent indeed gives different advantages, e.g., the possibility to exploit the EPR effect to achieve a passive targeting, the versatility of the possible modifications to improve their specificity, their responses to external stimuli exploitable for therapy, diagnosis or both purposes, and several others. Although the high number of published articles and millions of dollars invested in the development of new nanomedicines, only few proposals have demonstrated a real improvement compared to traditional protocols in terms of enhanced recovery rate and decrease of side effects [13]. Nowadays, a limited number of them are under clinical trials or approved, especially in the case of remotely triggered NPs intended as clinical agent without the addition of drugs. There are indeed several challenges to face to achieve the approval, as well as various biological, technological, and study design issues [206]. Actually, the properties of the NPs in the human body strictly influence their fate and therapeutic outcomes, and several chemical, physical, and biological methods to improve the NPs stability, their biomimetic properties and reduce their aggregation have been proposed [12]. The conjugation of the NPs with antibodies is particularly explored in the last years to achieve a specific tumor targeting. Another important aspect is the synthesis of the NPs, that strongly tailor the NPs behavior [207] as well has to be modulable for the industrial scale-up, and moreover the product of the synthesis, i.e., the NPs obtained, should be reproducible and must satisfy high standards before to be subjected to clinical trials and commercialized [206].

New systems to improve the drug delivery in particular have been tested and clinically approved either by the Food and Drug Administration (FDA) or the European Medicines Agency (EMA) in the last years.

Focusing on the anticancer therapy, in 1995 the FDA approved Doxil, composed by PEGylated liposomal doxorubicin, able to improve the doxorubin bioavailability. Several other liposomal formulations with the aim to increment the drug delivery have been developed, with the exception of Abraxane, that is composed by albumin‐bound paclitaxel NPs. Apart from the delivery of conventional drugs, a similar strategy was also implemented for gene therapy for the treatment of several pathologies, and several clinical trials are ongoing [208].

There are some investigations about the involvement of remotely activated NPs as imaging tool or for cancer cell killing; however, very few of them involve NPs without the addition of drugs.

In the context of imaging, iron oxide NPs in particular have been largely employed as contrast agent for MRI. These NPs indeed are able to easily penetrate into tumor cells thanks to the EPR effect leading the direct monitoring of cancer cells. Different technologies involving these NPs as imaging tool are nowadays under investigation [27, 206, 208]. A slightly different nanoconstruct, constituted by silica NPs delivering a NIR fluorophore and an iodine radiolabeled targeting peptide, is under trial for the imaging of tumors and metastasis. Other micro/nanosized tools, generally based on microbubbles encapsulated in lipids or HSA, have otherwise been adopted as US contrast agent to increment the echogenicity of the tissue [208].

Focusing of remotely activated NPs for cancer cell killing, there are some proposals under investigation, as reported in Table 1. Very recently hafnium oxide NPs with a negatively charged phosphate coating (NBTXR3/Hensify) produced by Nanobiotix were approved as radiation therapy enhancers in the treatment of soft-tissue sarcoma because of their capability to specifically amplify the radiation effects [39, 208]. The number of clinical trials concerning its involvement in the treatment of other cancers, evaluating different administration routes, and even a possible combination with immunotherapy has grown in the last years, and now include the treatment of head and neck cancer (NCT01946867, NCT02901483), liver cancer (NCT02721056), prostate cancer (NCT02805894), various kind of metastases (NCT03589339), soft tissue sarcoma (NCT02379845), rectal cancer (NCT02465593), pancreatic cancer (NCT04484909), lung non-small cell carcinoma (NCT04505267) [208].

**Table 1:** Clinical trials involving remotely triggered NPs for cancer cell death. Adapted from Ref. [208]

Name (company)	NP type	Stimulus	Disease	Clinical trial number and status
NBTXR3 PEP503 (Nanobiotix)	Hafnium oxide NPs	Radiation therapy	Solid primary tumors or metastasis	NCT01433068: completed NCT01946867: unknown NCT02901483: recruiting NCT02721056: unknown NCT02805894: recruiting NCT03589339: recruiting NCT02379845: unknown NCT02465593: recruiting NCT04484909: recruiting NCT04505267: not yet recruiting
AuroLase (nanospectra biosciences)	PEG-coated silica gold nanoshells	Near-infrared light	Solid primary and/or metastatic tumors	NCT01679470: terminated NCT02680535: active, not recruiting NCT00848042: completed NCT04240639: recruiting
Magnablate	Iron oxide NPs	Magnetic field	Prostate cancer	NCT02033447: completed

Another example is represented by pegylated silica gold nanoshells, that have been proposed, but not yet approved, in combination with NIR (AuroLase therapy-Nanospectra Bioscience) for the treatment of prostate cancer (NCT02680535) [209], primary and metastatic lung tumors (NCT01679470), head and neck tumor (NCT00848042). The nanosystem proposed indeed is able to accumulate in the tumor area, where causes thermal ablation upon a NIR irradiation. They have demonstrated to possess a low long-term toxicity, and their activation through an MRI/US guided laser irradiation is under evaluation (NCT04240639) [210].

Iron oxide NPs (Magnablate) have also been proposed to achieve thermal ablation under a MF stimulation in prostate cancer. These magnetic NPs can precisely accumulate into the target region through a direct injection and generate heat when a MF is applied. Additionally, the eventual extravasation of the NPs has to be non-toxic for the neighboring tissues. The aim of the clinical trial NCT02033447 is to test the localization of the NPs in patients prior to the surgical removal of the prostate without heating. However, the results of such trial are still unknown [27, 208].

There are other proposals involving nanosystems remotely activated by an external input, e.g., thermoresponsive liposomes, but the therapeutic agent is represented by a drug and has been revised elsewhere [27, 208].

## Conclusions

Cancer nanomedicine represents a promising strategy to achieve the complete tumor remission overcoming the limitations of the conventional anticancer approaches. To further improve the therapeutic outcomes, an innovative strategy consists in the adoption of NPs remotely activated through an external physical stimulation to trigger cancer cell death. Both the components involved, actually the NPs and the stimulation, are administrated in a non-toxic dose, and only in the tumor area they work synergistically maximizing the therapeutic efficacy and reducing the side effects for the adjacent healthy tissues.

Several possible combinations have nowadays been proposed, employing NPs constituted by a single material or hybrid tools, activated by one or more external physical inputs for therapy and/or diagnosis. This synergism is able to trigger NPs toxicity, and moreover NPs could contribute in several ways to improve the efficacy of the physical input, releasing other forms of energy (e.g., heat), and often contributing to improve its focalization. NPs besides protect healthy tissues, helping to focalize the therapeutic action only in the tumor area, where they actively contribute to maximize tumor ablation, and sometimes directly protecting the tissues surrounding the area of interest.

Anyway, as presented in the previous section about the clinical trials involving remotely activated NPs, only few of the proposals have reached the clinical phase. It has to be pointed out that we have considered only the cases where the therapeutic agent is represented by a NP triggering cell toxicity in synergy with a physical stimulation, thus without the involvement of other factors (i.e., drugs). Additionally, from Table 1 it is clear that the clinical trials actually available are related to only one stimulation per NP type.

This issue could be overcome by paying close attention to the following challenges that need to be approached in a multidisciplinary way. (1) The NP synthesis is strictly important to determine NPs features, it has to be scalable for the industrial production and reproducible. (2) A careful control over the physical, chemical properties of the NPs, as well as the evaluation of their behavior in different biological fluids is necessary, because these factors can dramatically tailor the therapeutic outcome. (3) It is mandatory to evaluate the NPs selectivity toward cancer cells with respect to the healthy ones. (4) The working mechanism of the synergy is a key parameter to understand how the therapy works and it could be ameliorated. (5) The passage from in vitro to in vivo is crucial to evaluate NPs fate in the body and the effectiveness of the therapy, and even the models should be carefully chosen with a scrupulous study-design.

Besides, the passage from research to clinic and industry appears to be nowadays still challenging and limited to few cases, even if there is a large number of possible combinations between physical stimulations and NPs that demonstrated an increased cytotoxic potential in vitro and in vivo. Therefore, many future efforts are needed to bring the excellent results of the research reported so far to a real application.

## Abbreviations

CTAB: Cetyltrimethylammonium bromide
DOPC: 1,2-Dioleoyl-sn-glycero-3-phosphocholine
EGFR: Epidermal growth factor receptor
EMA: European Medicines Agency
EPR: Enhanced permeability and retention effect
FDA: Food and Drug Administration
HSA: Human serum albumin
MF: Magnetic field
MFH: Magnetic fluid hyperthermia
MHT: Magnetic hyperthermia therapy
MRI: Magnetic resonance imaging
MW: Microwave
NaRFA: Nano-radio-frequency ablation
NIR: Near-infrared region
NMH: Nanomagnetic hyperthermia
NPs: Nanoparticles
PDT: Photodynamic therapy
PEG: Polyethylene glycol
PSi NWs: Porous silicon nanowires
PTT: Photothermal therapy
RF: Radiofrequency
ROS: Reactive oxygen species
SAR: Specific absorption rate
SDT: Sonodynamic therapy
SPR: Surface plasmon resonance
TA: Thermoacoustic
ZnO NCs: Zinc oxide nanocrystals
ZnO NPs: Zinc oxide nanoparticles

## References

[1] International Agency for Research in Cancer (2018). Latest Global Cancer Data.

[2] Hanahan D, Weinberg RA (2011). Hallmarks of cancer: the next generation. Cell.

[3] Tran S, DeGiovanni P-J, Piel B, Rai P (2017). Cancer nanomedicine: a review of recent success in drug delivery. Clin. Transl. Med..

[4] Soares S, Sousa J, Pais A, Vitorino C (2018). Nanomedicine: principles, properties, and regulatory issues. Front. Chem..

[5] Shi J, Kantoff PW, Wooster R, Farokhzad OC (2017). Cancer nanomedicine: progress, challenges and opportunities. Nat. Rev..

[6] Yan C, Guo Z, Shen Y, Chen Y, Tian H, Zhu WH (2018). Molecularly precise self-assembly of theranostic nanoprobes within a single-molecular framework for: in vivo tracking of tumor-specific chemotherapy. Chem. Sci..

[7] Dong P, Rakesh KP, Manukumar HM, Mohammed YHE, Karthik CS (2019). Innovative nano-carriers in anticancer drug delivery—a comprehensive review. Bioorg. Chem..

[8] Zhang J, Wang Q, Liu J, Guo Z, Yang J (2019). Saponin-based near-infrared nanoparticles with aggregation-induced emission behavior: enhancing cell compatibility and permeability. ACS Appl. Bio Mater..

[9] Ji X, Wang C, Tang M, Guo D, Peng F (2018). Biocompatible protamine sulfate@silicon nanoparticle-based gene nanocarriers featuring strong and stable fluorescence. Nanoscale.

[10] Racca L, Canta M, Dumontel B, Ancona A, Limongi T (2018). Zinc oxide nanostructures in biomedicine. Smart Nanoparticles Biomed..

[11] De Matteis V, Cascione M, Toma CC, Leporatti S (2018). Silver nanoparticles: synthetic routes, in vitro toxicity and theranostic applications for cancer disease. Nanomaterials.

[12] Limongi T, Canta M, Racca L, Ancona A, Tritta S, Vighetto V, Cauda V (2019). Improving dispersal of therapeutic nanoparticles in the human body. Nanomedicine.

[13] Youn YS, Bae YH (2018). Perspectives on the past, present, and future of cancer nanomedicine. Adv. Drug Deliv. Rev..

[14] Albanese A, Tang PS, Chan WCW (2012). The effect of nanoparticle size, shape, and surface chemistry on biological systems. Annu. Rev. Biomed. Eng..

[15] Sukhanova A, Bozrova S, Sokolov P, Berestovoy M, Karaulov A, Nabiev I (2018). Dependence of nanoparticle toxicity on their physical and chemical properties. Nanoscale Res. Lett..

[16] Canavese G, Ancona A, Racca L, Canta M, Dumontel B (2018). Nanoparticle-assisted ultrasound: a special focus on sonodynamic therapy against cancer. Chem. Eng. J..

[17] Kwatra D, Venugopal A, Anant S (2013). Nanoparticles in radiation therapy: a summary of various approaches to enhance radiosensitization in cancer. Transl. Cancer Res..

[18] Chang D, Lim M, Goos JACM, Qiao R, Ng YY (2018). Biologically targeted magnetic hyperthermia: potential and limitations. Front. Pharmacol..

[19] Zhang R, Yan F, Chen Y (2018). Exogenous physical irradiation on titania semiconductors: materials chemistry and tumor-specific nanomedicine. Adv. Sci..

[20] Collins CB, McCoy RS, Ackerson BJ, Collins GJ, Ackerson CJ (2014). Radiofrequency heating pathways for gold nanoparticles. Nanoscale.

[21] McWilliams B, Wang H, Binns V, Curto S, Bossmann S, Prakash P (2017). Experimental investigation of magnetic nanoparticle-enhanced microwave hyperthermia. J. Funct. Biomater..

[22] Yang Z, Sun Z, Ren Y, Chen X, Zhang W (2019). Advances in nanomaterials for use in photothermal and photodynamic therapeutics (review). Mol. Med. Rep..

[23] Vines JB, Yoon JH, Ryu NE, Lim DJ, Park H (2019). Gold nanoparticles for photothermal cancer therapy. Front. Chem..

[24] Howard D, Sebastian S, Le QVC, Thierry B, Kempson I (2020). Chemical mechanisms of nanoparticle radiosensitization and radioprotection: a review of structure-function relationships influencing reactive oxygen species. Int. J. Mol. Sci..

[25] Racca L, Limongi T, Vighetto V, Dumontel B, Ancona A (2020). Zinc oxide nanocrystals and high-energy shock waves: a new synergy for the treatment of cancer cells. Front. Bioeng. Biotechnol..

[26] Xiang H, Chen Y (2019). Energy-converting nanomedicine. Small.

[27] Parchur AK, Jagtap JM, Sharma G, Gogineni V, White SB, Joshi A (2019). Remotely triggered nanotheranostics. Nanotheranostics.

[28] Sneider A, Vandyke D, Paliwal S, Rai P (2017). Remotely triggered nano-theranostics for cancer applications. Nanotheranostics.

[29] Fan W, Yung B, Huang P, Chen X (2017). Nanotechnology for multimodal synergistic cancer therapy. Chem. Rev..

[30] Pizzino G, Irrera N, Cucinotta M, Pallio G, Mannino F, Arcoraci V, Squadrito F, Altavilla D, Bitto A (2017). Oxidative stress: harms and benefits for human health. Oxid. Med. Cell Longev..

[31] Vighetto V, Ancona A, Racca L, Limongi T, Troia A, Canavese G, Cauda V (2019). The synergistic effect of nanocrystals combined with ultrasound in the generation of reactive oxygen species for biomedical applications. Front. Bioeng. Biotechnol..

[32] Fan W, Tang W, Lau J, Shen Z, Xie J, Shi J, Chen X (2019). Breaking the depth dependence by nanotechnology-enhanced x-ray-excited deep cancer theranostics. Adv. Mater..

[33] Verry C, Sancey L, Dufort S, Le Duc G, Mendoza C (2019). Treatment of multiple brain metastases using gadolinium nanoparticles and radiotherapy: NANO-RAD, a phase I study protocol. BMJ Open.

[34] Deng J, Xu S, Hu W, Xun X, Zheng L, Su M (2018). Tumor targeted, stealthy and degradable bismuth nanoparticles for enhanced X-ray radiation therapy of breast cancer. Biomaterials.

[35] Kuncic Z, Lacombe S (2018). Nanoparticle radio-enhancement: principles, progress and application to cancer treatment. Phys. Med. Biol..

[36] Zhao J, Zhou M, Li C (2016). Synthetic nanoparticles for delivery of radioisotopes and radiosensitizers in cancer therapy. Cancer Nanotechnol..

[37] Retif P, Pinel S, Toussaint M, Frochot C, Chouikrat R, Bastogne T, Barberi-Heyob M (2015). Nanoparticles for radiation therapy enhancement: the key parameters. Theranostics.

[38] Liu Y, Zhang P, Li F, Jin X, Li J, Chen W, Li Q (2018). Metal-based nanoenhancers for future radiotherapy: radiosensitizing and synergistic effects on tumor cells. Theranostics.

[39] Bonvalot S, Rutkowski PL, Thariat J, Carrère S, Ducassou A (2019). NBTXR3, a first-in-class radioenhancer hafnium oxide nanoparticle, plus radiotherapy versus radiotherapy alone in patients with locally advanced soft-tissue sarcoma (Act. In. Sarc): a multicentre, phase 2–3, randomised, controlled trial. Lancet Oncol..

[40] Hauser AK, Mitov MI, Daley EF, McGarry RC, Anderson KW, Hilt JZ (2016). Targeted iron oxide nanoparticles for the enhancement of radiation therapy. Biomaterials.

[41] Chen F, Zhang XH, Hu XD, Liu PD, Zhang HQ (2018). The effects of combined selenium nanoparticles and radiation therapy on breast cancer cells in vitro. Artif. Cells Nanomed. Biotechnol..

[42] Meyer TJ, Scherzad A, Moratin H, Gehrke TE, Killisperger J (2019). The radiosensitizing effect of zinc oxide nanoparticles in sub-cytotoxic dosing is associated with oxidative stress in vitro. Materials.

[43] Xie J, Wang N, Dong X, Wang C, Du Z (2019). Graphdiyne nanoparticles with high free radical scavenging activity for radiation protection. ACS Appl. Mater. Interfaces..

[44] Abdi Goushbolagh N, Farhood B, Astani A, Nikfarjam A, Kalantari M, Zare MH (2018). Quantitative cytotoxicity, cellular uptake and radioprotection effect of cerium oxide nanoparticles in MRC-5 normal cells and MCF-7 cancerous cells. Bionanoscience.

[45] Gao Y, Chen K, Ma JL, Gao F (2014). Cerium oxide nanoparticles in cancer. Onco Targets Ther..

[46] Abbasi AZ, Gordijo CR, Amini MA, Maeda A, Rauth AM, DaCosta RS, Wu XY (2016). Hybrid manganese dioxide nanoparticles potentiate radiation therapy by modulating tumor hypoxia. Cancer Res..

[47] Durante M, Orecchia R, Loeffler JS (2017). Charged-particle therapy in cancer: clinical uses and future perspectives. Nat. Rev. Clin. Oncol..

[48] Lacombe S, Porcel E, Scifoni E (2017). Particle therapy and nanomedicine: state of art and research perspectives. Cancer Nanotechnol..

[49] Symonds P, Jones GDD (2019). FLASH radiotherapy: the next technological advance in radiation therapy?. Clin. Oncol..

[50] Degiovanni A, Amaldi U (2015). History of hadron therapy accelerators. Phys. Med..

[51] Khot MI, Andrew H, Svavarsdottir HS, Armstrong G, Quyn AJ, Jayne DG (2019). A review on the scope of photothermal therapy-based nanomedicines in preclinical models of colorectal cancer. Clin. Colorectal Cancer.

[52] A. Bettaieb, P.K. Wrzal, D.A. Averill-Bates, Hyperthermia: Cancer Treatment and Beyond. Cancer treatment-conventional and innovative approaches. 10.5772/55795

[53] Wang J, Qiu J (2016). A review of organic nanomaterials in photothermal cancer therapy. Cancer Res. Front..

[54] Lubner MG, Brace CL, Hinshaw JL, Lee FT (2010). Microwave tumor ablation: mechanism of action, clinical results and devices. J. Vasc. Interv. Radiol..

[55] Zhang B, Moser MAJ, Zhang EM, Luo Y, Liu C, Zhang W (2016). A review of radiofrequency ablation: large target tissue necrosis and mathematical modelling. Phys. Med..

[56] Barajas M, Fraga T, Acevedo M, Cabrera RG (2018). Radiofrequency ablation: a review of current knowledge, therapeutic perspectives, complications, and contraindications. Int. J. Biosens. Bioelectron..

[57] Pantano P, Harrison CD, Poulose J, Urrabazo D, Norman TQ (2017). Factors affecting the 13.56-MHz radio-frequency-mediated heating of gold nanoparticles. Appl. Spectrosc. Rev..

[58] Beyk J, Tavakoli H (2019). Selective radiofrequency ablation of tumor by magnetically targeting of multifunctional iron oxide-gold nanohybrid. J. Cancer Res. Clin. Oncol..

[59] Beik J, Abed Z, Ghoreishi FS, Hosseini-Nami S, Mehrzadi S, Shakeri-Zadeh A, Kamrava SK (2016). Nanotechnology in hyperthermia cancer therapy: from fundamental principles to advanced applications. J. Control. Release.

[60] Das P, Colombo M, Prosperi D (2019). Recent advances in magnetic fluid hyperthermia for cancer therapy. Colloids Surf. B Biointerfaces.

[61] Sharma SK, Shrivastava N, Rossi F, Tung LD, Thanh NTK (2019). Nanoparticles-based magnetic and photo induced hyperthermia for cancer treatment. Nano Today.

[62] Dulińska-Litewka J, Łazarczyk A, Hałubiec P, Szafrański O, Karnas K, Karewicz A (2019). Superparamagnetic iron oxide nanoparticles-current and prospective medical applications. Materials.

[63] Ashikbayeva Z, Tosi D, Balmassov D, Schena E, Saccomandi P, Inglezakis V (2019). Application of nanoparticles and nanomaterials in thermal ablation therapy of cancer. Nanomaterials.

[64] Hemery G, Genevois C, Couillaud F, Lacomme S, Gontier E (2017). Monocore: vs. multicore magnetic iron oxide nanoparticles: uptake by glioblastoma cells and efficiency for magnetic hyperthermia. Mol. Syst. Des. Eng..

[65] Kafrouni L, Savadogo O (2016). Recent progress on magnetic nanoparticles for magnetic hyperthermia. Prog. Biomater..

[66] Kalia S, Kango S, Kumar A, Haldorai Y, Kumari B, Kumar R (2014). Magnetic polymer nanocomposites for environmental and biomedical applications. Colloid Polym. Sci..

[67] Lavorato G, Lima E, Vasquez Mansilla M, Troiani H, Zysler R, Winkler E (2018). Bifunctional CoFe_2_O_4_/ZnO core/shell nanoparticles for magnetic fluid hyperthermia with controlled optical response. J. Phys. Chem. C.

[68] Jadhav SV, Shewale PS, Shin BC, Patil MP, Kim GD (2019). Study of structural and magnetic properties and heat induction of gadolinium-substituted manganese zinc ferrite nanoparticles for in vitro magnetic fluid hyperthermia. J. Colloid Interface Sci..

[69] Coşkun M, Korkmaz M (2014). The effect of SiO_2_ shell thickness on the magnetic properties of ZnFe_2_O_4_ nanoparticles. J. Nanoparticle Res..

[70] León Félix L, Sanz B, Sebastián V, Torres TE, Sousa MH (2019). Gold-decorated magnetic nanoparticles design for hyperthermia applications and as a potential platform for their surface-functionalization. Sci. Rep..

[71] Wu K, Su D, Liu J, Saha R, Wang JP (2019). Magnetic nanoparticles in nanomedicine: a review of recent advances. Nanotechnology.

[72] Hatamie S, Parseh B, Ahadian MM, Naghdabadi F, Saber R, Soleimani M (2018). Heat transfer of PEGylated cobalt ferrite nanofluids for magnetic fluid hyperthermia therapy: in vitro cellular study. J. Magn. Magn. Mater..

[73] Kandasamy G, Sudame A, Luthra T, Saini K, Maity D (2018). Functionalized hydrophilic superparamagnetic iron oxide nanoparticles for magnetic fluid hyperthermia application in liver cancer treatment. ACS Omega.

[74] Price PM, Mahmoud WE, Al-Ghamdi AA, Bronstein LM (2018). Magnetic drug delivery: where the field is going. Front. Chem..

[75] Cardinal J, Klune JR, Chory E, Jeyabalan G, Kanzius JS, Nalesnik M, Geller DA (2008). Non-invasive radiofrequency ablation of cancer targeted by gold nanoparticles. Surgery.

[76] Amini SM, Kharrazi S, Rezayat SM, Gilani K (2018). Radiofrequency electric field hyperthermia with gold nanostructures: role of particle shape and surface chemistry. Artif. Cells Nanomed. Biotechnol..

[77] Shao YL, Arjun B, Leo HL, Chua KJ (2017). Nano-assisted radiofrequency ablation of clinically extracted irregularly-shaped liver tumors. J. Therm. Biol.

[78] Gannon CJ, Cherukuri P, Yakobson BI, Cognet L, Kanzius JS (2007). Carbon nanotube-enhanced thermal destruction of cancer cells in a noninvasive radiofrequency field. Cancer.

[79] Bijukumar D, Girish CM, Sasidharan A, Nair S, Koyakutty M (2015). Transferrin-conjugated biodegradable graphene for targeted radiofrequency ablation of hepatocellular carcinoma. ACS Biomater. Sci. Eng..

[80] Raniszewski G, Miaskowski A, Wiak S (2015). The application of carbon nanotubes in magnetic fluid hyperthermia. J. Nanomater..

[81] Wu H, Liu G, Wang X, Zhang J, Chen Y (2011). Solvothermal synthesis of cobalt ferrite nanoparticles loaded on multiwalled carbon nanotubes for magnetic resonance imaging and drug delivery. Acta Biomater..

[82] Singh R, Torti SV (2013). Carbon nanotubes in hyperthermia therapy. Adv. Drug Deliv. Rev..

[83] Saghatchi F, Mohseni-Dargah M, Akbari-Birgani S, Saghatchi S, Kaboudin B (2020). Cancer therapy and imaging through functionalized carbon nanotubes decorated with magnetite and gold nanoparticles as a multimodal tool. Appl. Biochem. Biotechnol..

[84] Tamarov KP, Osminkina LA, Zinovyev SV, Maximova KA, Kargina JV (2014). Radio frequency radiation-induced hyperthermia using Si nanoparticle-based sensitizers for mild cancer therapy. Sci. Rep..

[85] Gongalsky M, Gvindzhiliia G, Tamarov K, Shalygina O, Pavlikov A (2019). Radiofrequency hyperthermia of cancer cells enhanced by silicic acid ions released during the biodegradation of porous silicon nanowires. ACS Omega.

[86] Ashokan A, Somasundaram VH, Gowd GS, Anna IM, Malarvizhi GL (2017). Biomineral nano-theranostic agent for magnetic resonance image guided, augmented radiofrequency ablation of liver tumor. Sci. Rep..

[87] Glazer ES, Curley SA (2011). Non-invasive radiofrequency ablation of malignancies mediated by quantum dots, gold nanoparticles and carbon nanotubes. Ther. Deliv..

[88] Glazer ES, Curley SA (2010). Radiofrequency field-induced thermal cytotoxicity in cancer cells treated with fluorescent nanoparticles. Cancer.

[89] Sidoff L, Dupuy DE (2017). Clinical experiences with microwave thermal ablation of lung malignancies. Int. J. Hyperth..

[90] Kim C (2018). Understanding the nuances of microwave ablation for more accurate post-treatment assessment. Future Oncol..

[91] Tan L, Tang W, Liu T, Ren X, Fu C (2016). Biocompatible hollow polydopamine nanoparticles loaded ionic liquid enhanced tumor microwave thermal ablation in vivo. ACS Appl. Mater. Interfaces..

[92] Beckler B, Cowan A, Farrar N, Murawski A, Robinson A (2018). Microwave Heating of antibody-functionalized carbon nanotubes as a feasible cancer treatment. Biomed. Phys. Eng. Exp..

[93] Wen L, Ding W, Yang S, Xing D (2016). Microwave pumped high-efficient thermoacoustic tumor therapy with single wall carbon nanotubes. Biomaterials.

[94] Wen L, Yang S, Zhong J, Zhou Q, Xing D (2017). Thermoacoustic imaging and therapy guidance based on ultra-short pulsed microwave pumped thermoelastic effect induced with superparamagnetic iron oxide nanoparticles. Theranostics.

[95] Jelbuldina M, Korobeinyk A, Korganbayev S, Tosi D, Dukenbayev K, Inglezakis VJ (2018). Real-time temperature monitoring in liver during magnetite nanoparticle-enhanced microwave ablation with fiber bragg grating sensors: ex vivo analysis. IEEE Sens. J..

[96] Tang T, Xu X, Wang Z, Tian J, Yang Y, Ou C, Bao H, Liu T (2019). Cu_2_ZnSnS_4_ nanocrystals for microwave thermal and microwave dynamic combination tumor therapy. Chem. Commun..

[97] Peng H, Ouyang J, Peng Y (2017). A simple approach for the synthesis of bi-functional Fe_3_O_4_@WO_3-x_ core–shell nanoparticles with magnetic-microwave to heat responsive properties. Inorg. Chem. Commun..

[98] Paudel NR, Shvydka D, Parsai EI (2016). A novel property of gold nanoparticles: free radical generation under microwave irradiation. Med. Phys..

[99] Kioko B, Ogundolie T, Adebiyi M, Ettinoffe Y, Rhodes C (2014). De-crystallization of uric acid crystals in synovial fluid using gold colloids and microwave heating. Nano Biomed. Eng..

[100] McLemore GL, Toker S, Boone-Kukoyi Z, Ajifa H, Lansiquot C (2017). Microwave heating of crystals with gold nanoparticles and synovial fluid under synthetic skin patches. ACS Omega.

[101] Ghahremani FH, Sazgarnia A, Bahreyni-Toosi MH, Rajabi O, Aledavood A (2011). Efficacy of microwave hyperthermia and chemotherapy in the presence of gold nanoparticles: an in vitro study on osteosarcoma. Int. J. Hyperth..

[102] Moradpoor R, Aledavood SA, Rajabi O, Chamani JK, Sazgarnia A (2017). Enhancement of cisplatin efficacy by gold nanoparticles or microwave hyperthermia? An in vitro study on a melanoma cell line. Int. J. Cancer Manag..

[103] Chu X, Mao L, Johnson O, Li K, Phan J, Zhang Y (2019). Exploration of TiO_2_ nanoparticle mediated microdynamic therapy on cancer treatment. Nanomed. Nanotechnol. Biol. Med..

[104] Wang S, Mei XG, Goldberg SN, Ahmed M, Lee JC (2016). Does thermosensitive liposomal vinorelbine improve end-point survival after percutaneous radiofrequency ablation of liver tumors in a mouse model?. Radiology.

[105] Wu S, Zhang D, Yu J, Dou J, Li X, Mu M, Liang P (2020). Chemotherapeutic nanoparticle-based liposomes enhance the efficiency of mild microwave ablation in hepatocellular carcinoma therapy. Front. Pharmacol..

[106] Dou JP, Wu Q, Fu CH, Zhang DY, Yu J, Meng XW, Liang P (2019). Amplified intracellular Ca^2+^ for synergistic anti-tumor therapy of microwave ablation and chemotherapy. J. Nanobiotechnol..

[107] Doughty ACV, Hoover AR, Layton E, Murray CK, Howard EW, Chen WR (2019). Nanomaterial applications in photothermal therapy for cancer. Materials.

[108] Liang J, Liu H, Yu J, Zhou L, Zhu J (2019). Plasmon-enhanced solar vapor generation. Nanophotonics.

[109] Kim M, Lee JH, Nam JM (2019). Plasmonic photothermal nanoparticles for biomedical applications. Adv. Sci..

[110] Wei W, Zhang X, Zhang S, Wei G, Su Z (2019). Biomedical and bioactive engineered nanomaterials for targeted tumor photothermal therapy: a review. Mater. Sci. Eng., C.

[111] Yang W, Liang H, Ma S, Wang D, Huang J (2019). Gold nanoparticle based photothermal therapy: development and application for effective cancer treatment. Sustain. Mater. Technol..

[112] Ali MRK, Wu Y, El-Sayed MA (2019). Gold-nanoparticle-assisted plasmonic photothermal therapy advances toward clinical application. J. Phys. Chem. C.

[113] Sancho-Albero M, Navascués N, Mendoza G, Sebastián V, Arruebo M, Martín-Duque P, Santamaría J (2019). Exosome origin determines cell targeting and the transfer of therapeutic nanoparticles towards target cells. J. Nanobiotechnol..

[114] Estelrich J, Antònia Busquets M (2018). Iron oxide nanoparticles in photothermal therapy. Molecules.

[115] Lu GH, Shang WT, Deng H, Han ZY, Hu M (2019). Targeting carbon nanotubes based on IGF-1R for photothermal therapy of orthotopic pancreatic cancer guided by optical imaging. Biomaterials.

[116] Mou J, Lin T, Huang F, Chen H, Shi J (2016). Black titania-based theranostic nanoplatform for single NIR laser induced dual-modal imaging-guided PTT/PDT. Biomaterials.

[117] Vines JB, Lim DJ, Park H (2018). Contemporary polymer-based nanoparticle systems for photothermal therapy. Polymers.

[118] Wang J, Yan R, Guo F, Yu M, Tan F, Li N (2016). Targeted lipid-polyaniline hybrid nanoparticles for photoacoustic imaging guided photothermal therapy of cancer. Nanotechnology.

[119] Dos Santos AF, De Almeida DRQ, Terra LF, Baptista MS, Labriola L (2019). Photodynamic therapy in cancer treatment—an update review. J. Cancer Metastasis Treat.

[120] Mallidi S, Anbil S, Bulin AL, Obaid G, Ichikawa M, Hasan T (2016). Beyond the barriers of light penetration: strategies, perspectives and possibilities for photodynamic therapy. Theranostics.

[121] van Straten D, Mashayekhi V, de Bruijn HS, Oliveira S, Robinson DJ (2017). Oncologic photodynamic therapy: basic principles, current clinical status and future directions. Cancers.

[122] Li X, Lovell JF, Yoon J, Chen X (2020). Clinical development and potential of photothermal and photodynamic therapies for cancer. Nat. Rev. Clin. Oncol..

[123] Baskaran R, Lee J, Yang S-G (2018). Clinical development of photodynamic agents and therapeutic applications. Biomater. Res..

[124] Lismont M, Dreesen L, Wuttke S (2017). Metal-organic framework nanoparticles in photodynamic therapy: current status and perspectives. Adv. Funct. Mater..

[125] Lucky SS, Soo KC, Zhang Y (2015). Nanoparticles in photodynamic therapy. Chem. Rev..

[126] Lu D, Tao R, Wang Z (2019). Carbon-based materials for photodynamic therapy: a mini-review. Front. Chem. Sci. Eng..

[127] Bogdan J, Pławińska-Czarnak J, Zarzyńska J (2017). Nanoparticles of titanium and zinc oxides as novel agents in tumor treatment: a review. Nanoscale Res. Lett..

[128] Youssef Z, Vanderesse R, Colombeau L, Baros F, Roques-Carmes T (2017). The application of titanium dioxide, zinc oxide, fullerene, and graphene nanoparticles in photodynamic therapy. Cancer Nanotechnol..

[129] Grebinyk A, Grebinyk S, Prylutska S, Ritter U, Matyshevska O, Dandekar T, Frohme M (2018). C 60 fullerene accumulation in human leukemic cells and perspectives of LED-mediated photodynamic therapy. Free Radic. Biol. Med..

[130] Ancona A, Dumontel B, Garino N, Demarco B, Chatzitheodoridou D (2018). Lipid-coated zinc oxide nanoparticles as innovative ROS-generators for photodynamic therapy in cancer cells. Nanomaterials.

[131] Rehman FU, Zhao C, Jiang H, Wang X (2016). Biomedical applications of nano-titania in theranostics and photodynamic therapy. Biomater. Sci..

[132] Ziental D, Czarczynska-Goslinska B, Mlynarczyk DT, Glowacka-Sobotta A, Stanisz B, Goslinski T, Sobotta L (2020). Titanium dioxide nanoparticles: prospects and applications in medicine. Nanomaterials.

[133] Ni W, Li M, Cui J, Xing Z, Li Z (2017). 808 nm light triggered black TiO_2_ nanoparticles for killing of bladder cancer cells. Mater. Sci. Eng., C.

[134] Yi C, Yu Z, Ren Q, Liu X, Wang Y (2020). Nanoscale ZnO-based photosensitizers for photodynamic therapy. Photodiagnosis Photodyn. Ther..

[135] Gupta J, Bahadur D (2017). Visible light sensitive mesoporous cu-substituted zno nanoassembly for enhanced photocatalysis, bacterial inhibition, and noninvasive tumor regression. ACS Sustain. Chem. Eng..

[136] Sivasubramanian M, Chuang YC, Lo LW (2019). Evolution of nanoparticle-mediated photodynamic therapy: from superficial to deep-seated cancers. Molecules.

[137] Zhang C, Zhao K, Bu W, Ni D, Liu Y, Feng J, Shi J (2015). Marriage of scintillator and semiconductor for synchronous radiotherapy and deep photodynamic therapy with diminished oxygen dependence. Angew. Chem. Int. Ed..

[138] Calixto GMF, Bernegossi J, De Freitas LM, Fontana CR, Chorilli M, Grumezescu AM (2016). Nanotechnology-based drug delivery systems for photodynamic therapy of cancer: a review. Molecules.

[139] Yi G, Hong SH, Son J, Yoo J, Park C, Choi Y, Koo H (2018). Recent advances in nanoparticle carriers for photodynamic therapy. Quant. Imaging Med. Surg..

[140] Yang G, Xu L, Chao Y, Xu J, Sun X (2017). Hollow MnO_2_ as a tumor-microenvironment-responsive biodegradable nano-platform for combination therapy favoring antitumor immune responses. Nat. Commun..

[141] Shibaguchi H, Tsuru H, Kuroki M, Kuroki M (2011). Sonodynamic cancer therapy: a non-invasive and repeatable approach using low-intensity ultrasound with a sonosensitizer. Anticancer Res..

[142] Izadifar Z, Babyn P, Chapman D (2017). Mechanical and biological effects of ultrasound: a review of present knowledge. Ultrasound Med. Biol..

[143] Xu H, Zhang X, Han R, Yang P, Ma H (2016). Nanoparticles in sonodynamic therapy: state of the art review. RSC Adv..

[144] Wan GY, Liu Y, Chen BW, Liu YY, Wang YS, Zhang N (2016). Recent advances of sonodynamic therapy in cancer treatment. Cancer Biol. Med..

[145] Sviridov AP, Osminkina LA, Nikolaev AL, Kudryavtsev AA, Vasiliev AN, Timoshenko VY (2015). Lowering of the cavitation threshold in aqueous suspensions of porous silicon nanoparticles for sonodynamic therapy applications. Appl. Phys. Lett..

[146] Osminkina LA, Nikolaev AL, Sviridov AP, Andronova NV, Tamarov KP (2015). Porous silicon nanoparticles as efficient sensitizers for sonodynamic therapy of cancer. Microporous Mesoporous Mater..

[147] Seil JT, Webster TJ (2012). Antibacterial effect of zinc oxide nanoparticles combined with ultrasound. Nanotechnology.

[148] Ebrahimi Fard A, Zarepour A, Zarrabi A, Shanei A, Salehi H (2015). Synergistic effect of the combination of triethylene-glycol modified Fe_3_O_4_ nanoparticles and ultrasound wave on MCF-7 cells. J. Magn. Magn. Mater..

[149] Marino A, Battaglini M, De Pasquale D, Degl’Innocenti A, Ciofani G (2018). Ultrasound-activated piezoelectric nanoparticles inhibit proliferation of breast cancer cells. Sci. Rep..

[150] Lafond M, Yoshizawa S, Ichiro Umemura S (2019). Sonodynamic therapy: advances and challenges in clinical translation. J. Ultrasound Med..

[151] Wang X, Chen H, Zheng Y, Ma M, Chen Y (2013). Au-nanoparticle coated mesoporous silica nanocapsule-based multifunctional platform for ultrasound mediated imaging, cytoclasis and tumor ablation. Biomaterials.

[152] Brazzale C, Canaparo R, Racca L, Foglietta F, Durando G (2016). Enhanced selective sonosensitizing efficacy of ultrasound-based anticancer treatment by targeted gold nanoparticles. Nanomedicine.

[153] Bernard V, Mornstein V, Jaroš J, Sedláčková M, Škorpíková J (2014). Combined effect of silver nanoparticles and therapeutical ultrasound on ovarian carcinoma cells A2780. J. Appl. Biomed..

[154] Han X, Huang J, Jing X, Yang D, Lin H (2018). Oxygen-deficient black titania for synergistic/enhanced sonodynamic and photoinduced cancer therapy at near infrared-II biowindow. ACS Nano.

[155] Dibaji SAR, Al-Rjoub MF, Myers MR, Banerjee RK (2013). Enhanced heat transfer and thermal dose using magnetic nanoparticles during HIFU thermal ablation-an in vitro study. J. Nanotechnol. Eng. Med..

[156] Kosheleva OK, Lai TC, Chen NG, Hsiao M, Chen CH (2016). Selective killing of cancer cells by nanoparticle-assisted ultrasound. J. Nanobiotechnol..

[157] Gong F, Cheng L, Yang N, Betzer O, Feng L (2019). Ultrasmall oxygen-deficient bimetallic oxide mnwox nanoparticles for depletion of endogenous gsh and enhanced sonodynamic cancer therapy. Adv. Mater..

[158] Sviridov A, Tamarov K, Fesenko I, Xu W, Andreev V, Timoshenko V, Lehto VP (2019). Cavitation induced by Janus-like mesoporous silicon nanoparticles enhances ultrasound hyperthermia. Front. Chem..

[159] Kharin A, Syshchyk O, Geloen A, Alekseev S, Rogov A, Lysenko V, Timoshenko V (2015). Carbon fluoroxide nanoparticles as fluorescent labels and sonosensitizers for theranostic applications. Sci. Technol. Adv. Mater..

[160] Pan X, Bai L, Wang H, Wu Q, Wang H (2018). Metal–organic-framework-derived carbon nanostructure augmented sonodynamic cancer therapy. Adv. Mater..

[161] Marino A, Almici E, Migliorin S, Tapeinos C, Battaglini M (2019). Piezoelectric barium titanate nanostimulators for the treatment of glioblastoma multiforme. J. Colloid Interface Sci..

[162] d’Agostino MC, Craig K, Tibalt E, Respizzi S (2015). Shock wave as biological therapeutic tool: from mechanical stimulation to recovery and healing, through mechanotransduction. Int. J. Surg..

[163] Foglietta F, Duchi S, Canaparo R, Varchi G, Lucarelli E, Dozza B, Serpe L (2017). Selective sensitiveness of mesenchymal stem cells to shock waves leads to anticancer effect in human cancer cell co-cultures. Life Sci..

[164] Marano F, Frairia R, Rinella L, Argenziano M, Bussolati B (2017). Combining doxorubicin-nanobubbles and shockwaves for anaplastic thyroid cancer treatment: preclinical study in a xenograft mouse model. Endocr. Relat. Cancer.

[165] Canaparo R, Serpe L, Zara GP, Chiarle R, Berta L, Frairia R (2008). High energy shock waves (HESW) increase paclitaxel efficacy in a syngeneic model of breast cancer. Technol. Cancer Res. Treat..

[166] Zhang J, Shrivastava S, Cleveland RO, Rabbitts TH (2019). Lipid-mRNA nanoparticle designed to enhance intracellular delivery mediated by shock waves. ACS Appl. Mater. Interfaces..

[167] López-Marín LM, Rivera AL, Fernández F, Loske AM (2018). Shock wave-induced permeabilization of mammalian cells. Phys. Life Rev..

[168] Canaparo R, Serpe L, Catalano MG, Bosco O, Zara GP, Berta L, Frairia R (2006). High energy shock waves (HESW) for sonodynamic therapy: effects on HT-29 human colon cancer cells. Anticancer Res..

[169] Serpe L, Canaparo R, Berta L, Bargoni A, Zara GP, Frairia R (2011). High energy shock waves and 5-aminolevulinic for sonodynamic therapy: effects in a syngeneic model of colon cancer. Technol. Cancer Res. Treat..

[170] Foglietta F, Canaparo R, Francovich A, Arena F, Civera S (2015). Sonodynamic treatment as an innovative bimodal anticancer approach: shock wave-mediated tumor growth inhibition in a syngeneic breast cancer model. Discov. Med..

[171] Varchi G, Foglietta F, Canaparo R, Ballestri M, Arena F (2015). Engineered porphyrin loaded core-shell nanoparticles for selective sonodynamic anticancer treatment. Nanomedicine.

[172] Canaparo R, Varchi G, Ballestri M, Foglietta F, Sotgiu G (2013). Polymeric nanoparticles enhance the sonodynamic activity of meso-tetrakis (4-sulfonatophenyl) porphyrin in an in vitro neuroblastoma model. Int. J. Nanomed..

[173] Wang L, Meng D, Hao Y, Zhao Y, Li D (2015). Gold nanostars mediated combined photothermal and photodynamic therapy and X-ray imaging for cancer theranostic applications. J. Biomater. Appl..

[174] Shanei A, Akbari-Zadeh H (2019). Investigating the sonodynamic-radiosensitivity effect of gold nanoparticles on HeLa cervical cancer cells. J. Korean Med. Sci..

[175] Behrouzkia Z, Joveini Z, Keshavarzi B, Eyvazzadeh N, Aghdam RZ (2016). Hyperthermia: how can it be used?. Oman Med. J..

[176] Jiang PS, Tsai HY, Drake P, Wang FN, Chiang CS (2017). Gadolinium-doped iron oxide nanoparticles induced magnetic field hyperthermia combined with radiotherapy increases tumour response by vascular disruption and improved oxygenation. Int. J. Hyperth..

[177] Li M, Zhao Q, Yi X, Zhong X, Song G (2016). Au@MnS@ZnS core/shell/shell nanoparticles for magnetic resonance imaging and enhanced cancer radiation therapy. ACS Appl. Mater. Interfaces..

[178] Ma N, Jiang YW, Zhang X, Wu H, Myers JN (2016). Enhanced radiosensitization of gold nanospikes via hyperthermia in combined cancer radiation and photothermal therapy. ACS Appl. Mater. Interfaces..

[179] Hainfeld JF, Lin L, Slatkin L, Avraham Dilmanian F, Vadas TM, Smilowitz HM (2014). Gold nanoparticle hyperthermia reduces radiotherapy dose. Nanomed. Nanotechnol. Biol. Med..

[180] Yu X, Li A, Zhao C, Yang K, Chen X, Li W (2017). Ultrasmall semimetal nanoparticles of bismuth for dual-modal computed tomography/photoacoustic imaging and synergistic thermoradiotherapy. ACS Nano.

[181] Daneshvar F, Salehi F, Karimi M, Vais RD, Mosleh-Shirazi MA, Sattarahmady N (2020). Combined X-ray radiotherapy and laser photothermal therapy of melanoma cancer cells using dual-sensitization of platinum nanoparticles. J. Photochem. Photobiol. B Biol..

[182] Zhou M, Chen Y, Adachi M, Wen X, Erwin B (2015). Single agent nanoparticle for radiotherapy and radio-photothermal therapy in anaplastic thyroid cancer. Biomaterials.

[183] Hosseini V, Mirrahimi M, Shakeri-Zadeh A, Koosha F, Ghalandari B (2018). Multimodal cancer cell therapy using Au@Fe_2_O_3_ core–shell nanoparticles in combination with photo-thermo-radiotherapy. Photodiagnosis Photodyn. Ther..

[184] Movahedi MM, Alamzadeh Z, Hosseini-Nami S, Shakeri-Zadeh A, Taheripak G (2020). Investigating the mechanisms behind extensive death in human cancer cells following nanoparticle assisted photo-thermo-radiotherapy. Photodiagnosis Photodyn. Ther..

[185] Liu J, Yang Y, Zhu W, Yi X, Dong Z (2016). Nanoscale metal-organic frameworks for combined photodynamic and radiation therapy in cancer treatment. Biomaterials.

[186] Western C, Hristov D, Schlosser J (2015). Ultrasound imaging in radiation therapy: from interfractional to intrafractional guidance. Cureus.

[187] Cirincione R, Di Maggio FM, Forte GI, Minafra L, Bravatà V (2017). High-intensity focused ultrasound- and radiation therapy-induced immuno-modulation: comparison and potential opportunities. Ultrasound Med. Biol..

[188] Liu X, Zhang Y, Wang Y, Zhu W, Li G (2020). Comprehensive understanding of magnetic hyperthermia for improving antitumor therapeutic efficacy. Theranostics.

[189] Espinosa A, Di Corato R, Kolosnjaj-Tabi J, Flaud P, Pellegrino T, Wilhelm C (2016). Duality of iron oxide nanoparticles in cancer therapy: amplification of heating efficiency by magnetic hyperthermia and photothermal bimodal treatment. ACS Nano.

[190] Ma X, Wang Y, Liu XL, Ma H, Li G (2019). Fe_3_O_4_-Pd Janus nanoparticles with amplified dual-mode hyperthermia and enhanced ROS generation for breast cancer treatment. Nanoscale Horiz..

[191] Di Corato R, Béalle G, Kolosnjaj-Tabi J, Espinosa A, Clément O (2015). Combining magnetic hyperthermia and photodynamic therapy for tumor ablation with photoresponsive magnetic liposomes. ACS Nano.

[192] Curcio A, Silva AKA, Cabana S, Espinosa A, Baptiste B (2019). Iron oxide nanoflowers@CuS hybrids for cancer tri-therapy: interplay of photothermal therapy, magnetic hyperthermia and photodynamic therapy. Theranostics.

[193] Józefczak A, Kaczmarek K, Hornowski T, Kubovčíková M, Rozynek Z, Timko M, Skumiel A (2016). Magnetic nanoparticles for enhancing the effectiveness of ultrasonic hyperthermia. Appl. Phys. Lett..

[194] Beguin E, Gray MD, Logan KA, Nesbitt H, Sheng Y (2020). Magnetic microbubble mediated chemo-sonodynamic therapy using a combined magnetic-acoustic device. J. Control Release.

[195] Xiao S, Hu Z, He Y, Jin H, Yang Y (2020). Enhancement effect of microbubble-enhanced ultrasound in microwave ablation in rabbit VX_2_ liver tumors. Biomed. Res. Int..

[196] Zhou Z, Wang Y, Song S, Wu W, Wu S, Tsui PH (2019). Monitoring microwave ablation using ultrasound echo decorrelation imaging: an ex vivo study. Sensors.

[197] Gebreel D, Shalaby T, Yousef Y, Mohamed M, Badawy H (2014). Magnetic fluid based on Fe_3_O_4_ nanoparticles: preparation and hyperthermia application. Int. J. Chem. Appl. Biol. Sci..

[198] Li X, Liu Y, Fu F, Cheng M, Liu Y (2019). Single NIR laser-activated multifunctional nanoparticles for cascaded photothermal and oxygen-independent photodynamic therapy. Nano-Micro Lett..

[199] Luo S, Yang Z, Tan X, Wang Y, Zeng Y (2016). Multifunctional photosensitizer grafted on polyethylene glycol and polyethylenimine dual-functionalized nanographene oxide for cancer-targeted near-infrared imaging and synergistic phototherapy. ACS Appl. Mater. Interfaces..

[200] Yao C, Zhang L, Wang J, He Y, Xin J (2016). Gold nanoparticle mediated phototherapy for cancer. J. Nanomater..

[201] Li Q, Hong L, Li H, Liu C (2017). Graphene oxide-fullerene C60 (GO-C60) hybrid for photodynamic and photothermal therapy triggered by near-infrared light. Biosens. Bioelectron..

[202] Lee J, Lee YH, Jeong CB, Choi JS, Chang KS, Yoon M (2018). Gold nanorods-conjugated TiO_2_ nanoclusters for the synergistic combination of phototherapeutic treatments of cancer cells. J. Nanobiotechnol..

[203] Sazgarnia A, Shanei A, Taheri AR, Tayyebi Meibodi N, Eshghi H, Attaran N, Shanei M (2013). The therapeutic effect of acoustic cavitation on breast carcinoma tumor model in BALB/c mice in the presence of gold nanoparticles. J. Ultrasound Med..

[204] Dai C, Zhang S, Liu Z, Wu R, Chen Y (2017). Two-dimensional graphene augments nanosonosensitized sonocatalytic tumor eradication. ACS Nano.

[205] Gao F, He G, Yin H, Chen J, Liu Y (2019). Titania-coated 2D gold nanoplates as nanoagents for synergistic photothermal/sonodynamic therapy in the second near-infrared window. Nanoscale.

[206] Anselmo AC, Mitragotri S (2016). Nanoparticles in the clinic. Bioeng. Transl. Med..

[207] Garino N, Limongi T, Dumontel B, Canta M, Racca L (2019). A microwave-assisted synthesis of zinc oxide nanocrystals finely tuned for biological applications. Nanomaterials.

[208] Anselmo AC, Mitragotri S (2019). Nanoparticles in the clinic: an update. Bioeng. Transl. Med..

[209] Stern JM, Kibanov Solomonov VV, Sazykina E, Schwartz JA, Gad SC, Goodrich GP (2016). Initial evaluation of the safety of nanoshell-directed photothermal therapy in the treatment of prostate disease. Int. J. Toxicol..

[210] Rastinehad AR, Anastos H, Wajswol E, Winoker JS, Sfakianos JP (2019). Gold nanoshell-localized photothermal ablation of prostate tumors in a clinical pilot device study. Proc. Natl. Acad. Sci. U.S.A..

