# Correction to: Remotely Activated Nanoparticles for Anticancer Therapy

**DOI:** 10.1007/s40820-020-00553-8

**Published:** 2020-11-20

**Authors:** Luisa Racca, Valentina Cauda

**Affiliations:** Department of Applied Science and Technology, Politecnico di Torino, C.so Duca degli Abruzzi 24, 10129 Turin, Italy

## Correction to: Nano-Micro Lett. (2021) 13:11 10.1007/s40820-020-00537-8

In the original publication figures 7 and 11 need be updated with correct values. The correct version of Figs. 7 and 11 is provided in this correction. The original article has been corrected.

**Fig. 7:**
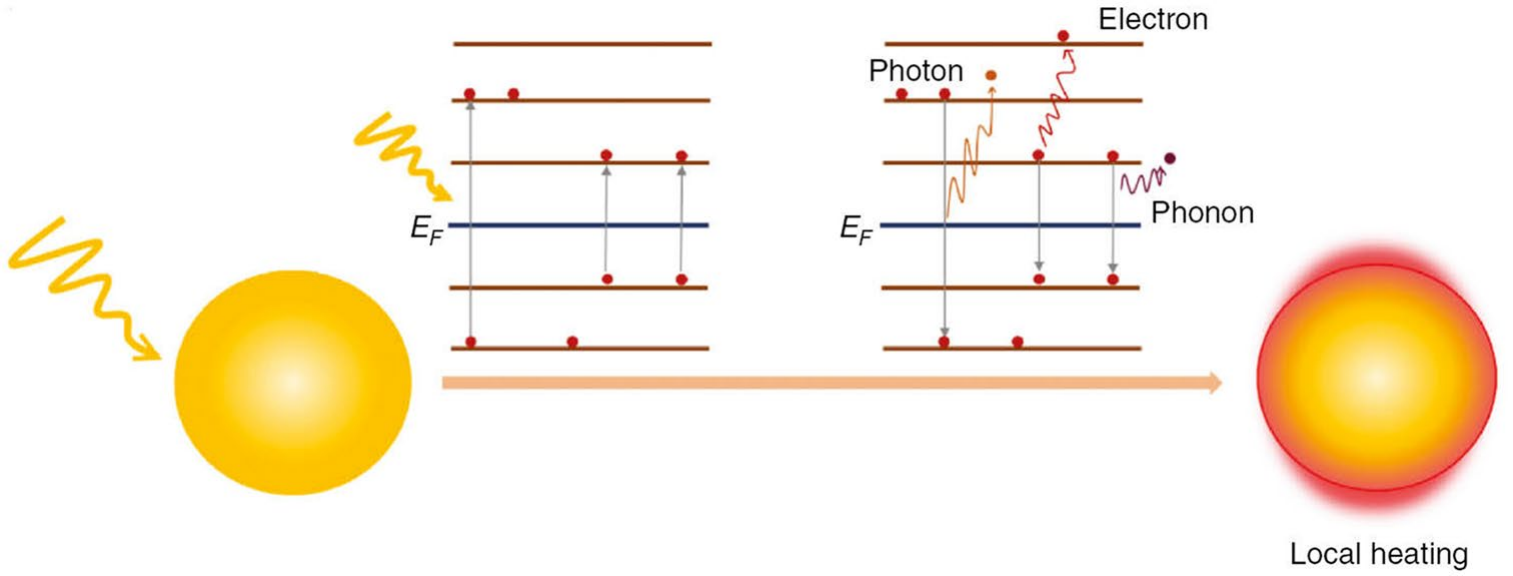
PTT mechanism. Plasmon decay (electron-to-photon, electron-to-electron, and electron-to-phonon) generates local heating. Reprinted under a Creative Common Licence CC-BY 4.0. Copyright 2020 from Ref. [108]

**Fig. 11:**
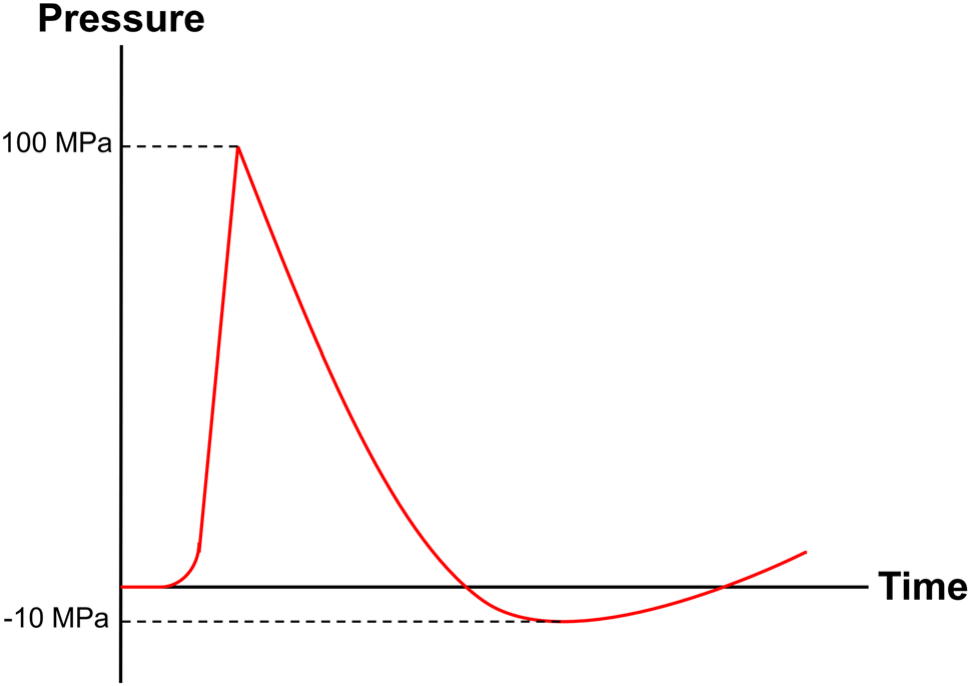
Scheme of a therapeutic SW

